# Impact of modified aggregate gradation on the workability, mechanical, microstructural and radiation shielding properties of recycled aggregate concrete

**DOI:** 10.1038/s41598-025-02655-y

**Published:** 2025-05-26

**Authors:** Mohy S. Fattouh, Mohamed A. Abouelnour, Alaa A. Mahmoud, Islam N. Fathy, A. F. El Sayed, Sameh A. Elhameed, Islam M. Nabil

**Affiliations:** 1 Civil Engineering Department, Faculty of Engineering, Sinai University, Arish, Egypt; 2 Civil Engineering Department, Faculty of Engineering, Fayoum University, Fayoum, Egypt; 3 Construction and Building Engineering Department, October High Institute for Engineering & Technology, Giza, Egypt; 4 Physics Department, Faculty of Science, Cairo University, Cairo, Egypt; 5 Chemical and Nuclear Engineering Department, Military Technical College, Kobry El-kobbah, Cairo, Egypt; 6 Physics Department, Faculty of Science, Fayoum University, Fayoum, Egypt

**Keywords:** Recycled concrete aggregate, Gradation, Slump, Mechanical properties, Radiation shielding, Engineering, Materials science, Physics

## Abstract

The increasing need for sustainable construction practices has driven research into the utilization of recycled concrete aggregate (RCA) in new concrete mixes. However, the variable properties of RCA, particularly its gradation, can significantly impact the performance of the resulting concrete. This study investigates the impact of modified aggregate gradation on the properties of concrete incorporating 100% RCA. Three concrete mixes were prepared: a control mix with the original RCA gradation (RCA1) and two modified mixes (RCA2 and RCA3). The initial gradation was modified by enhancing the 10% passing the #1″ sieve (S1) and diminishing the combined 10% passing the #1″ sieve and 10% passing the #4″ sieve (S2). Subsequently, the effects of these modified gradations were assessed, encompassing workability, mechanical properties (compressive and flexural strength), microstructural characteristics (XRD, SEM, and EDX), and radiation shielding performance. Results demonstrate that, despite reduced workability, the modified mixes exhibited enhanced compressive and flexural strengths. Microstructural analysis revealed denser, more interconnected cement paste matrices and refined interfacial transition zones (ITZ) in the modified mixes. Furthermore, the radiation shielding effectiveness increased with increasing gradation modification (RCA1 < RCA2 < RCA3). Specifically, RCA3 exhibited the lowest half-value layer (HV), tenth-value layer (TV), and mean free path (MF), along with the highest average macroscopic removal cross-section (FCS). These results suggest that modifying the gradation of RCA can effectively improve the mechanical and radiation shielding properties of concrete made with 100% RCA. This enhanced performance makes RCA concrete a viable option for radiation shielding applications in facilities like hospitals and nuclear power plants, contributing to both sustainable construction and improved safety.

**Supplementary Information:**

The online version contains supplementary material available at 10.1038/s41598-025-02655-y.

## Introduction

Devices that are capable of producing artificial ionizing radiation, such as X-rays and γ-rays, have been extensively utilized in a wide range of industrial, medical, and nuclear configurations. The utilization of technological advancements is directly responsible for this widespread adoption. On the other hand, prolonged and excessive exposure to such radiation can have a negative impact on health, potentially leading to the development of cancer, in addition to symptoms such as nausea, vomiting, and, in the most severe cases, death^1^. The interaction of high-energy photons with human tissue results in the ionization of water molecules within the tissue. Neutrons, γ-rays, and X-rays pose a threat to the environment, as well as to people and animals. For all of these reasons, the pursuit of discovering improved materials for radiation shielding/attenuation is something that a lot of researchers are interested in .

Sustainable construction practices are gaining prominence due to the growing emphasis on environmental stewardship. One aspect of sustainable construction involves the effective utilization of construction and demolition waste (C&D waste). C&D waste encompasses solid materials generated during the construction, renovation, or demolition of various structures, including residential, commercial, and infrastructure facilities^2^. Recycled concrete waste can be processed into recycled concrete aggregate (RCA)^3^, which has historically been utilized in lower-value applications such as land reclamation and subbase roads^4^. Using RCA offers a significant environmental advantage. By decreasing the need for new mining operations, RCA contribute to resource conservation and environmental sustainability^5^. This practice helps to reduce the ecological footprint of construction projects and minimize the negative impacts of mining activities, such as habitat destruction, water pollution, and greenhouse gas emissions^6^. Additionally, using RCA can reduce the overall carbon footprint of concrete production by diverting waste materials from landfills and reducing the energy consumption associated with manufacturing new aggregates^7^. Furthermore, incorporating RCA into concrete mixtures can provide economic benefits.

By utilizing waste materials, construction projects can potentially reduce material costs and contribute to a more sustainable and circular economy^8^. This can lead to cost savings for both contractors and building owners, ultimately promoting the adoption of more environmentally friendly construction practices. Recent advancements in waste management technology have facilitated the production of high-quality RCA comparable to natural aggregate. This has led to a surge in interest in using RCA as a structural component in high-value applications. Understanding the unique properties of RCA and its potential impact on concrete mixtures is essential for effective utilization of these recycled materials in construction applications. RCA is distinguished from conventional concrete by its unique material properties. RCA typically exhibit higher water absorption rates, lower specific gravity^9^, and greater porosity compared to natural aggregates^10^. The residual mortar adhered to RCA derived from recycled concrete waste further enhances their water absorption capacity^11^.

Research has indicated that natural aggregate exhibits significantly lower water absorption rates compared to RCA. Under saturated surface dry conditions, water absorption rates for concrete with natural aggregate were found to range from 0.5 to 1%, while RCA rates were between 4% and 4.7%, representing a difference of up to 4.2%^12^. Studies have found that RCAs absorb between 4.9% and 5.2% of their weight in water, while concrete with natural aggregate absorb only 1.0–2.5%^13^. This higher water absorption rate can have several implications for the use of RCA in concrete. For example, it can affect the workability of the concrete mixture, as well as its strength and durability. This substantial disparity in water absorption capacity is primarily attributed to the presence of microcracks and voids within the RCA, which can facilitate the infiltration of water. To address the subpar physical and mechanical properties of RCA, researchers have investigated various treatment methods. These methods focus on removing loose mortar particles from the aggregate surface and improving the quality of the mortar adhering to it^14^. Polymer treatments were found to significantly decrease water absorption^15^. Like regular concrete, RCA has a correlation between its strength and the water-to-cement ratio. Yet, RCA typically shows reduced strength because of microcracks caused by crushing and the weak or porous nature of the mortar used in the RCA^16^. To achieve the desired slump, increasing the paste content while maintaining a constant water-to-cement ratio is a more effective approach than solely adding water^17^. Concrete samples were prepared with RCA to assess its impact on the strength of high-strength concrete. The study showed that up to 30% RCA can be incorporated without major strength or durability reductions^13^.

Studies have demonstrated that employing a well-graded aggregate gradation with a maximum size of 20 mm can produce concrete with desirable properties when using either natural aggregate or recycled aggregate^18^. Modified aggregate gradation concrete refer to concrete specimens that have been created using a specific alteration to the standard aggregate size distribution. This modification is intended to optimize the concrete’s properties, such as strength, durability, and workability. This study examined the impact of using RCA derived from North Sinai demolished structures in concrete mixtures. Three mixtures were prepared: the original gradation, a modified aggregate gradation mix with 10% #1″ aggregate (S1), and a mix with 10% #1″, 10% #4″, and a 10% reduction in #3/8″ aggregate (S2). The concrete samples underwent a thorough evaluation, assessing their fresh and mechanical properties such as workability, compressive strength, and flexural strength. To analyse the microstructure and elemental composition, advanced techniques like X-ray diffraction (XRD), scanning electron microscopy (SEM), and energy dispersive X-ray spectroscopy (EDX) were employed. The radiation shielding properties of the RCAX concrete samples were evaluated by selecting those with the highest compressive strength. Monte Carlo simulations utilizing the MCNP algorithm and Phy-X software were conducted to compute several radiation shielding parameters, including linear attenuation (µ) and half value layer (HV), among others. The fast neutron cross-section, half-value layer (HVL_FCS_), and relaxation length (λ_FCS_) were computed utilizing the MCNP code.

This research holds significant implications for sustainable construction and advanced material applications. By investigating the impact of specific RCA gradation modifications on mechanical, microstructural, and, importantly, radiation shielding properties, this study addresses a critical need for high-value utilization of construction and demolition waste. Demonstrating the viability of 100% RCA concrete, particularly with tailored gradation, for radiation shielding applications offers a sustainable alternative to conventional materials in facilities like hospitals and nuclear power plants, contributing to resource conservation and reduced environmental impact. Furthermore, the observed improvements in mechanical properties and radiation shielding effectiveness resulting from the modified gradations offer a pathway to enhance the performance of RCA concrete, overcoming limitations associated with its inherent variability and expanding its potential applications. This work not only contributes to the fundamental understanding of RCA concrete behavior but also provides practical insights for developing sustainable and high-performance construction materials for specialized applications, ultimately promoting a circular economy within the construction sector. This research is thus novel in its holistic approach, examining the interplay between modified aggregate gradation, microstructure, and radiation shielding performance in 100% RCA concrete, offering valuable insights for developing sustainable radiation shielding materials.

## Materials and experimental study

### Materials properties

#### Cement

**Fig. 1:**
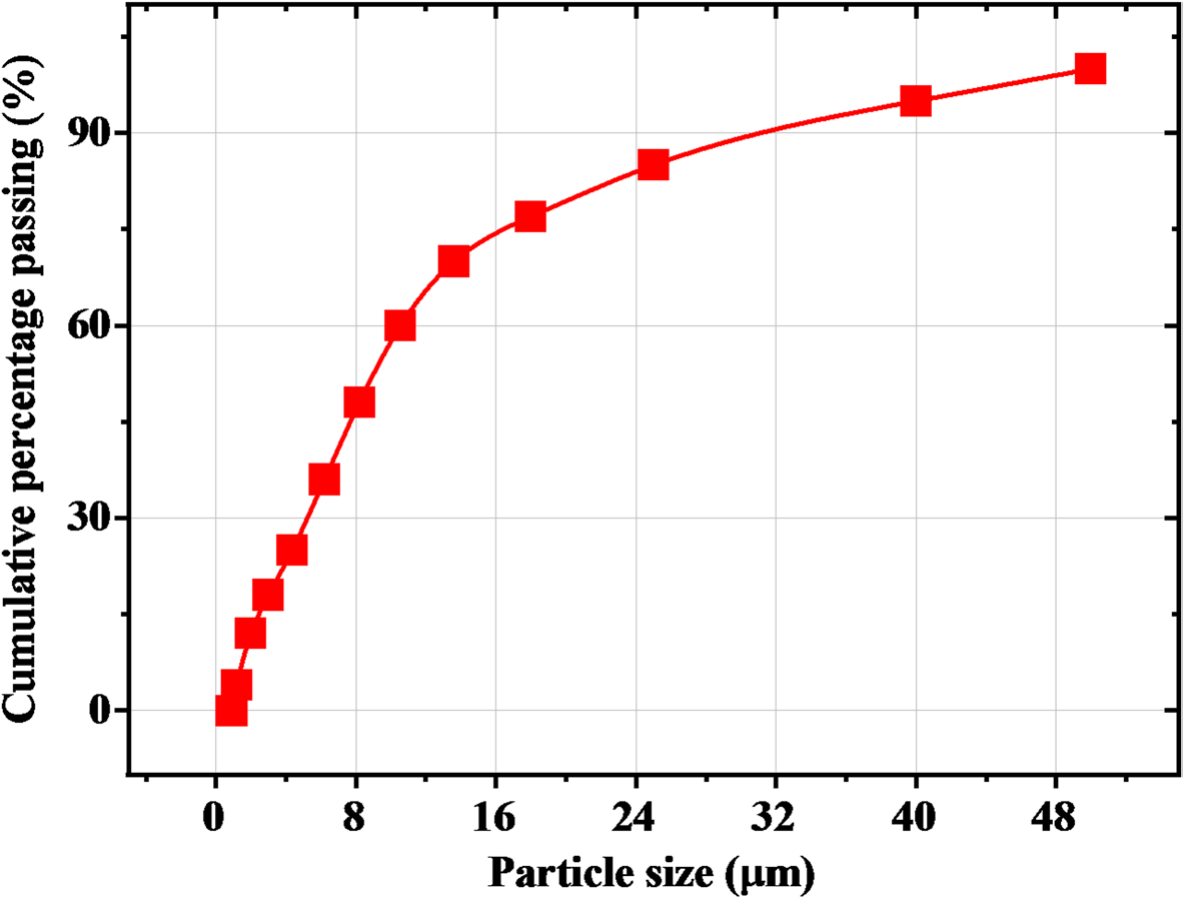
Particle size distribution of used cement.

Ordinary Portland cement produced by the local company Sinai cement factory was used in the study. This cement (CEM I 52.5 N), conforms to the ASTM C 150 standard and had an average compressive strength of 53 MPa after 28 days^19^. More details about the physical, mechanical, and chemical properties of the used cement are provided in Tables 1 and 2. The specific gravity of the cement sample was measured using a Le Chatelier flask according to ASTM C 188. Standard consistency, initial and final setting times were evaluated using a Vicat apparatus following established procedures. The specific surface area of cement is measured using the Blaine apparatus, and the relevant ASTM standard is indeed ASTM C204. Soundness, indicating resistance to post-hardening expansion, was assessed using a Le Chatelier apparatus according to ASTM C 1157. Compressive strength testing was performed after a 28-day curing period, adhering to ASTM C109/C109M-11 guidelines. X-Ray Fluorescence (XRF) analysis determined the cement’s chemical composition, a key factor in hydration kinetics and long-term performance. Additionally, the particle size distribution of the used cement is a relationship between the particle size and the cumulative percentage of particles passing as cleared in Fig. 1.

**Table 1:** Properties of used cement.

Physical properties					Soundness (mm)	Mortar compressive strength (MPa)		
Colour	Specific gravity	Initial setting time (min)	Final setting time (min)	Surface area (cm^2^/g)		3 days	7 days	28 days
Grey	3.14	60	360	3475	1	27.5	38.4	53

**Table 2:** Chemical composition of used cement (% mass).

SiO_2_	CaO	Fe_2_O_3_	Al_2_O_3_	SO_3_	MgO	Na_2_O	LOI*
19.27	62.19	1.75	3.87	2.8	2.18	0.2	3.15

*LOI: Loss on Ignition at 1000 °C.

#### Aggregate

**Fig. 2:**
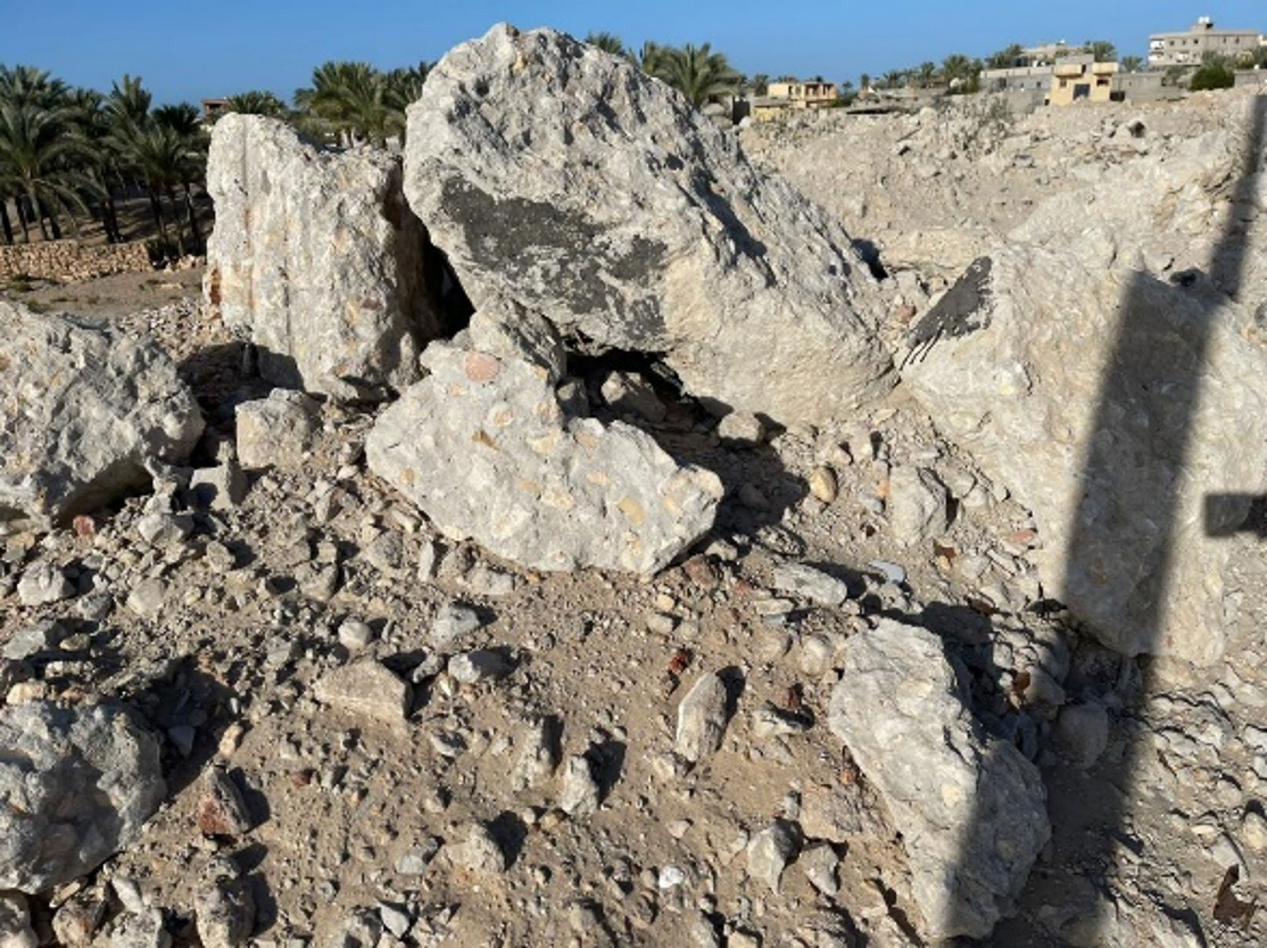
RCA from El-Arish, North Sinai.

**Fig. 3:**
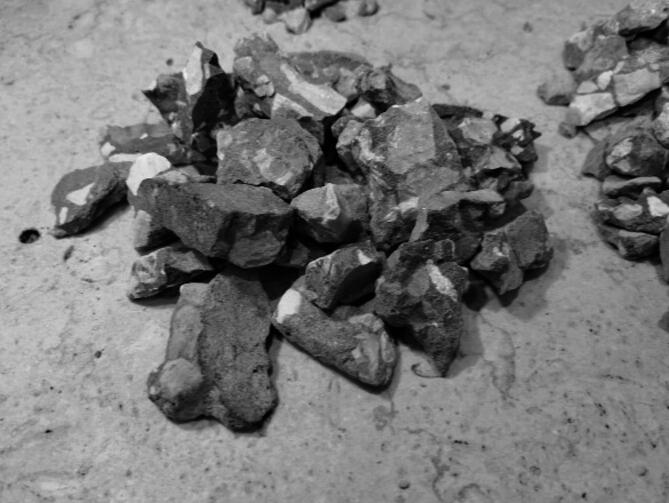
RCA after crushing.

In this particular investigation, the coarse aggregate that was utilized was obtained from quarries located in the El-Arish North Sinai Region. The RCA, shown in Fig. 2, exhibited a distinctive angular shape with a relatively smooth texture, reflecting the characteristics of the original materials. Many of the RCA particles were partially or completely covered by residual mortar, which softened their edges and overall appearance. Despite this, the RCA particles were carefully classified and graded according to ASTM C33 standards^20^. While most particles adhered to these standards, a small percentage of RCA exceeded the grading limits by a slight margin. To represent various sources and regions in North Sinai, recycled concrete aggregates (RCAs) were collected from discarded concrete. These RCAs were crushed using a jaw crusher to obtain recycled aggregates of suitable size and gradation (Fig. 3), which were then analyzed according to ASTM C136 methods^21^. The concrete was initially broken down with a steel hammer, then crushed into specific size ranges with a jaw crusher. The resulting material was screened using a vibrating sieve to ensure a maximum particle size of 20 millimeters. The concrete mix also included natural quartzite sand that had not been crushed. The natural sand that is obtained from the El-Arish quarry in North Sinai is perfect for use as fine aggregate because it has a particle size distribution that ranges from 0 to 4.75 millimeters. This used sand possesses the necessary characteristics to meet the requirements of ASTM C33. Table 3 presents gradation of used sand. Table 4 presents the physical properties of the various aggregate types used in the concrete mixtures investigated in this study which meet the standards. The particle size distribution of the used coarse aggregates within the upper and lower limits of the Egyptian code.

**Fig. 4:**
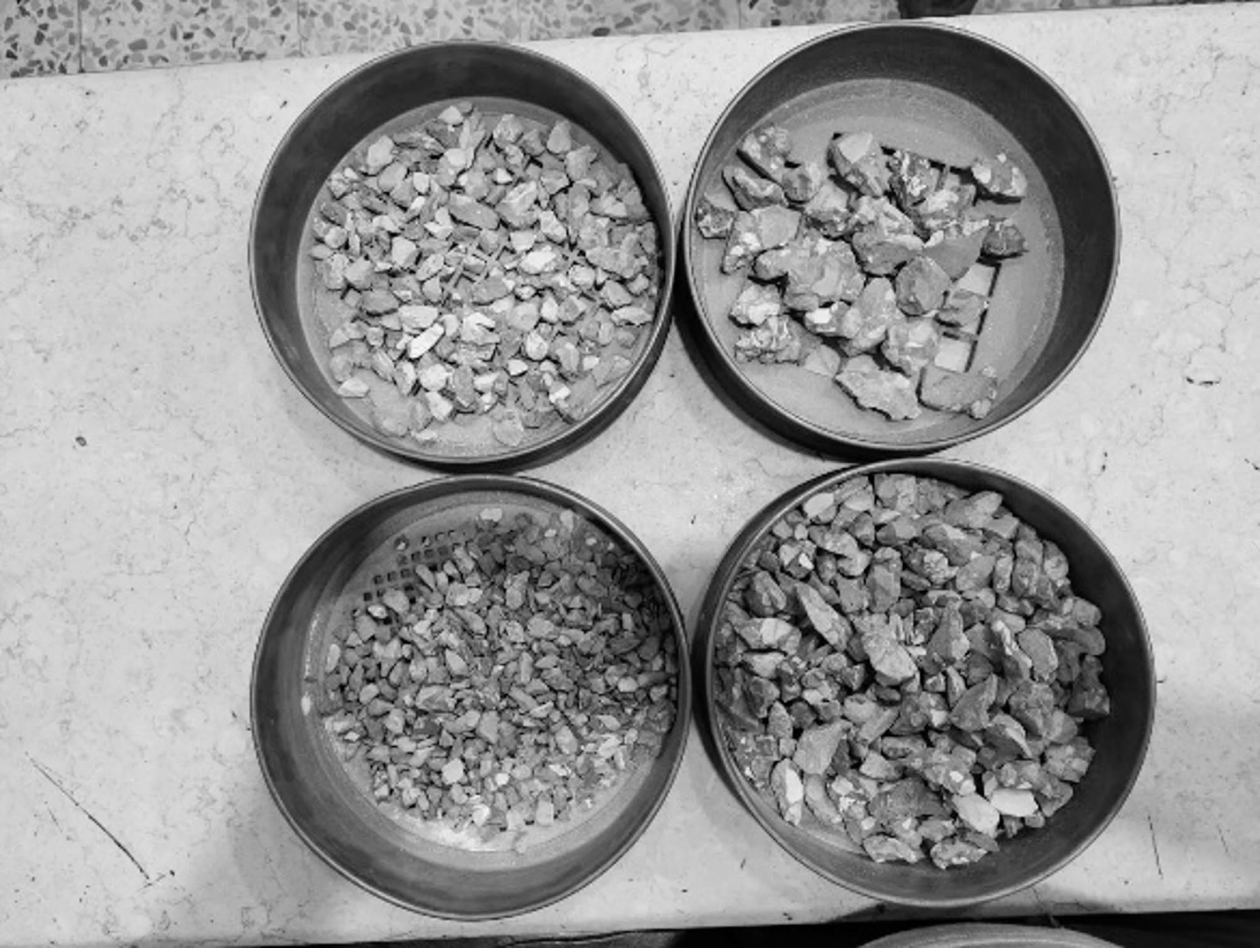
Sieve analysis test.

**Fig. 5:**
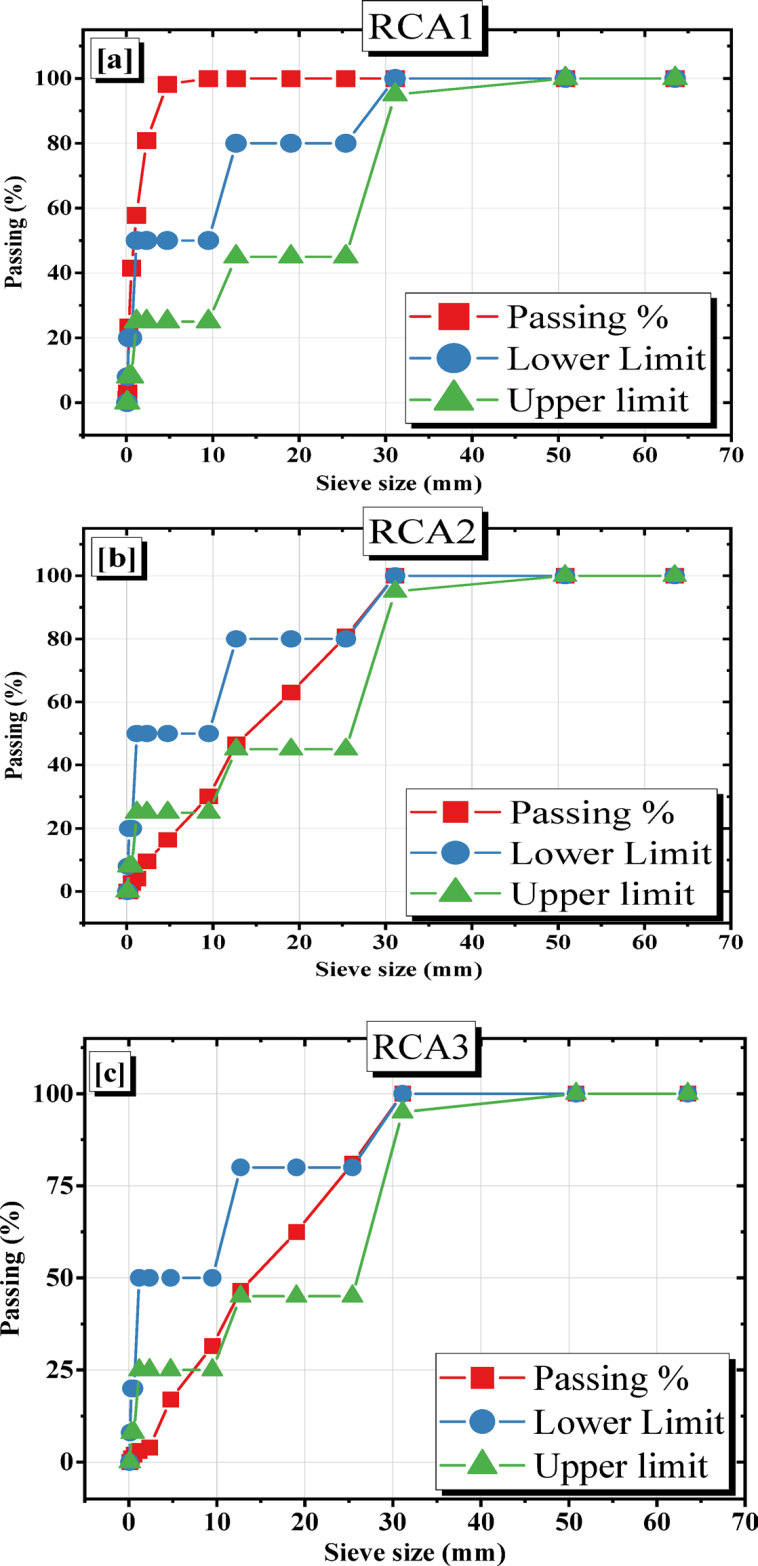
Distribution of the particle size for (**a**) RCA1, (**b**) RCA2, and (**c**) RCA3.

Table 5 details the variations in RCA gradation for each mixture. The control mix, RCA1, used the original aggregate gradation, while the other mixtures (RCA2 and RCA3) were modified to align with Tennessee Department of Transportation (TDOT) paving concrete practices. Recycled aggregate was conducted using sieve analysis test as displayed in Fig. 4 to get the gradation of RCA1 with the original aggregate gradation. RCA2 is a modified aggregate gradation mix with a 10% increase in the proportion of #1″ (S1). RCA3 is a modified aggregate gradation mix with a 10% increase in the proportions of both #1″ and #4″ aggregates, and a 10% decrease in the proportion of #3/8″ aggregate (S2). Table 6 displays the sieves analysis and the % deviation in gradations RCAs. Figure 5 displays the particle size distribution of the different gradations of aggregate in the mixtures.

**Table 3:** Gradation of sand.

Sieve size (mm)	9.5	4.75	2.36	1.18	0.6	0.3	0.15
ASTM C33 limits (%)	100	95–100	80–100	50–85	25–60	5–30	0–10
Passing %	100	100	97	75	55	25	2

**Table 4:** Physical properties of aggregates.

Physical properties	Sand	RCA	Standard
Water absorption %	1.3	2.7	ASTM C127
Bulk specific gravity	2.6	2.3	ASTM C127
SSD specific gravity	2.4	2.4	ASTM C127
Bulk density, kg/m^3^	1700	1580	ASTM C29
Apparent specific gravity	2.5	2.56	ASTM C127
Los Angles abrasion coefficient (%)	–	33.4	ASTM C131
Crushing value (%)	–	26.7	BS 812
Fineness modulus	2.46	–	ASTM C33

**Table 5:** The characteristics of the gradation of RCA.

Symbol	Content
RCA1	100% original gradation of RCA
RCA 2	10% #1” improved gradation (S1)
RCA 3	10% # 1” + 10% # 4” + reduction10% # 3/8” (S2)

**Table 6:** Sieves analysis and the % deviation in gradations RCA.

Sieve size (inch)	2 1/2″	2″	1 1/2″	1″	3/4″	1/2″	3/8″	#4	#8	#16	#30	#50	#100	#200
Egyptian code	100	100	95–100	45–80	45–80	45–80	25–50	25–50	25–50	25–50	8–20	8–20	0–8	0
RCA1	100.0	100.0	100.0	100.0	100.0	100.0	100.0	97.4	83.2	61.4	43.5	33.3	3.0	1.0
RCA2	100.0	100.0	100.0	80.8	63	47.5	30.5	15.4	11.41	5.4	4.3	0	0	0
RCA3	100.0	100.0	100.0	81	62.5	46.5	31.5	17	4	3	2	1	0	0

#### Water and superplasticizer

The concrete specimens were mixed and cured with clean, fresh water in accordance with Egyptian requirements for concrete design and construction. To enhance workability, a commercially available superplasticizer, “Sikament NN,” was added during mixing. This admixture, from the Egyptian branch of Sika Company, complies with ASTM C494^22^ standards for Type F admixtures and was used at a fixed rate of 2.35% of the water weight. The superplasticizer (SP) was incorporated due to the use of RCA with high absorption and a relatively low water-to-cement ratio, which could lead to reduced workability. Table 7 provides detailed information about the properties of the superplasticizer used.

**Table 7:** Used superplasticizer properties.

Optimum using ratio (% of water weight)	Structure of material	Density	Chlorine ratio	Alkaline ratio	Solid content ratio	Category
1–3%	Sulfonated naphthalene formaldehyde	1.12–1.25 kg/L	< 0.1%	< 3%	32%	High-range water reducer

### Mix proportions and methodology

Three distinct concrete mixes, each designed to produce 1 m^3^ of concrete, were prepared. The constituent quantities were held constant across all mixes, while the gradation of the RCA was systematically varied (Table 8). To establish a baseline, the control mix (RCA1) was formulated using 100% original gradation of RCA and adhered strictly to established criteria. The other two mixes (RCA2 and RCA3) consist of the same quantities of components with different in gradation as shown in the table. The mix quantities consisted of 350 kg of cement, 1210 kg of coarse aggregate, 755 kg of fine aggregate, and 170 kg of water. A constant water/cement (W/C) ratio of 0.486 was maintained for all mixtures, and a superplasticizer dosage of 2.35% by weight of the mixing water was consistently used to ensure consistent workability. By using a constant W/C ratio and SP, the study aimed to control the workability and strength properties of the concrete mixtures, allowing for a more focused evaluation of the impact of aggregate gradation on the overall performance. The use of a superplasticizer also helped to improve the workability of the concrete, making it easier to place and compact.

**Table 8:** Mix proportions (kg/m^3^).

Mix	RCA code	Cement	Sand	RCA quantity (gradation)	Water	SP
Mix 1	RCA1	350	755	1210 (100% original gradation of RCA)	170	4
Mix 2	RCA2	350	755	1210 (10% #1″ improved gradation (S1))	170	4
Mix 3	RCA3	350	755	1210 (10% # 1″ + 10% # 4″ + reduction10% # 3/8″ (S2) )	170	4

### Mixing, casting and curing of concrete specimens

To evaluate the influence of varying RCA gradations on compressive and flexural strength, concrete samples were meticulously prepared in standardized 100 × 100 × 100 mm steel cubes and 100 × 100 × 500 mm beams. A rigorous mixing process was followed to ensure consistency across all concrete mixtures. Dry concrete mixture components were sequentially blended in a mixer to achieve thorough distribution and prevent segregation. Coarse and fine aggregates were initially dry-mixed for one minute to ensure homogenous distribution and prevent clumping. Subsequently, cement was added and mixed for two minutes to facilitate complete integration and ensure proper bonding with the aggregates. Finally, pre-measured water quantities, containing a dissolved liquid SP, were introduced and mechanically mixed for an additional two minutes to guarantee complete dispersion and prevent air voids. Following the mixing process, the fresh concrete was carefully transferred to the molds and compacted using an electric vibrator to eliminate air voids and ensure a dense and uniform structure. The top surfaces were then troweled smooth to maintain consistent geometry and facilitate accurate testing. After one day of curing within the molds, the concrete specimens were carefully demolded and submerged in a clean water tank maintained at a constant temperature of 25 ± 2 °C and a relative humidity exceeding 95%. This controlled environment provided optimal conditions for hydration and strength development, minimizing the effects of temperature fluctuations and moisture loss. The specimens remained submerged until the designated testing times arrived, allowing for adequate curing and strength development.

### Testing procedures

#### Fresh, mechanical, and microstructural properties

Workability was evaluated using the standard slump cone test, adhering to ASTM C143^23^. The slump test measures the deformation of a cone-shaped concrete sample after it is removed from the slump cone, providing an indication of its workability. To evaluate compressive strength, cubic concrete samples measuring 100 × 100 × 100 mm were tested using a uniaxial loading instrument with a capacity of 3000 kN. For each mixture, three samples were tested at 7, 28, and 90 days of curing. The reported compressive strength is the average of these values.

To assess flexural strength, prismatic concrete beams measuring 100 × 100 × 500 mm were used. The flexural strength test, an indirect measure of tensile strength, was conducted on three specimens from each mixture after 28 days of curing. The average flexural strength was calculated. A four-point bending test, was performed on all prismatic samples. Load was applied at a constant rate until failure. The crushing load was recorded, and flexural strength was calculated for each sample.

To analyze the microstructure properties of the concrete specimens, XRD, SEM, and EDX analyses were conducted. XRD was used to examine the characteristics and microstructure of the cement paste samples. For XRD measurements, the samples were crushed into a uniform powder passing from 75-micron sieve to be ready for testing and analyzed using an X’Pert Pro diffractometer. SEM analysis was performed on the concrete specimens to verify the microstructure properties and the mechanisms predicted by other tests. The concrete specimens were scanned with a narrow electron beam, and the resulting X-ray photons were detected by a silicon detector. The used device model was attached with EDX unit. EDX is a valuable analytical tool for determining the elemental composition of concrete samples, providing quantitative data on the relative amounts of various elements within a sample.

#### Radiation-shielding investigation

**Fig. 6:**
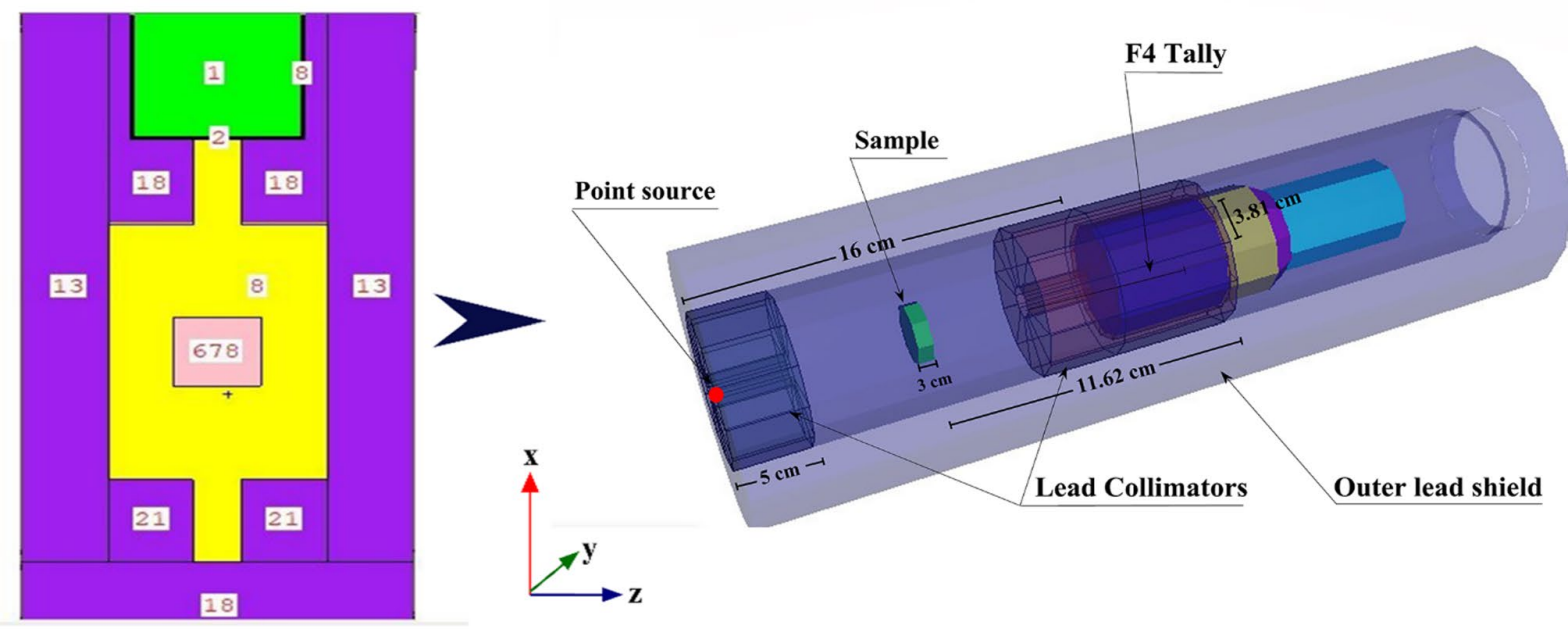
3D view of the radiation simulation system used for the RCAX samples.

The Monte Carlo for nature particles (MCNP) is a general code which uses the Monte Carlo method to simulate real-world gamma, and neutrons radiation. The purpose was to compare the γ-particle/waves intensity before and after traversing the examined RCA concrete materials. Radiation safety and shielding, dose computation, detector design, and many research domains often prefer MCNP codes. The appeal for these codes is attributed to their several advantageous properties, including rapid calculations, flexibility to various geometrical configurations, and functionality throughout a broad spectrum of energy levels. This strategy considers many photon interaction pathways and seeks to enhance the mobility of neutrons, and γ-rays. The MCNP simulation system was utilized to determine the theoretical effect of γ-rays and neutron particles emitted by any volume/point sources on any material^24^. In order to run an MCNP simulation, precise data about the concrete samples being studied is required, including their densities, chemical composition, and elemental makeup, in addition to their dimensions, source-to-detector distance, and geometry^25^. The simulation’s geometric configuration was developed based on a preset two and three-dimensional structure, as illustrated in Fig. 6. Within the experimental setup, all parameters have been found to be consistent^26^. To run the MCNP simulation, the input files needed to be created were made using text lines. There were distinct parts to the cell: a radioactive source, a detector, a cubic sample, a collimator for γ-radiations, and a cell itself. A mono γ-energetic flow from a source card was recognized as a point source of γ-rays for each input file at 0.015 ≤ Pγ < 15 MeV during the analysis. The neutron source is identified as a californium spectrum, operating within the En ≤ 11 MeV range to facilitate quick elimination of σ attenuation. The densities and elemental compositions of the inspected specimens were recorded in the material card of the text lines. Simulated γ/neutron radiation sources can have their mean duration of γ-rays and neutrons computed simultaneously using the F4:P/N tally. For the computations, an Intel Core i5 was used. Numerous NPS (11.5^7^) tries were conducted for each individual file to ensure that the random statistical errors were under 1%. The PhyX software was employed to corroborate the simulation and calculated results. A variables’ shielding parameters (e.g., µ, µ_c_, etc.) should be calculated to know the performance of any material^27^, the appropriate mathematical equations and definitions used in this study were as follows^28^:

1 $${\text{I}}\,=\,{{\text{I}}_{\text{o}}}{{\text{e}}^{ - \mu {\text{x}}}}$$

2 $$\:{\upmu\:}_\text{c}=\frac{\mu\:}{\rho\:}$$

3 $$\:\text{H}\text{V}=\:\frac{\text{l}\text{n}2}{{\upmu\:}}\:\:\:\:$$

4 $$\:\text{T}\text{V}=\:3.32\:HV_{1/2}$$

5 $$\:\text{M}\text{F}=\:\frac{1}{{\upmu\:}}\:\:\:\:$$

The radiation protection (RP) is a crucial metric to consider when assessing the extent to which different shielding materials can reduce radiation as follows^29^:

6 $$\:RP,{\%}=\left(1-\frac{I}{{I}_{o}}\right)100$$

The neutron decrease potential of suggested RCAX may be investigated by calculating the fast neutron removal (FCS) using the equation FCS =$\:\sum\:_{i}{W}_{i}$ x ρ. The symbol $\:{W}_{i}\:$represents the partial density, ρ represents the material density, and the subscript i represents the mass cross-section (σ) of the component)^30^. The half-value layer (HVL_FCS_) was determined using the formula. $\:{HVL}_{\text{F}\text{C}\text{S}}\:=\:\frac{ln2}{FCS}$, and the relaxation length (λ_FCS_) was calculated as $\:\frac{1}{FCS}$^ 31^.

## Results and discussion

### Slump cone

**Fig. 7:**
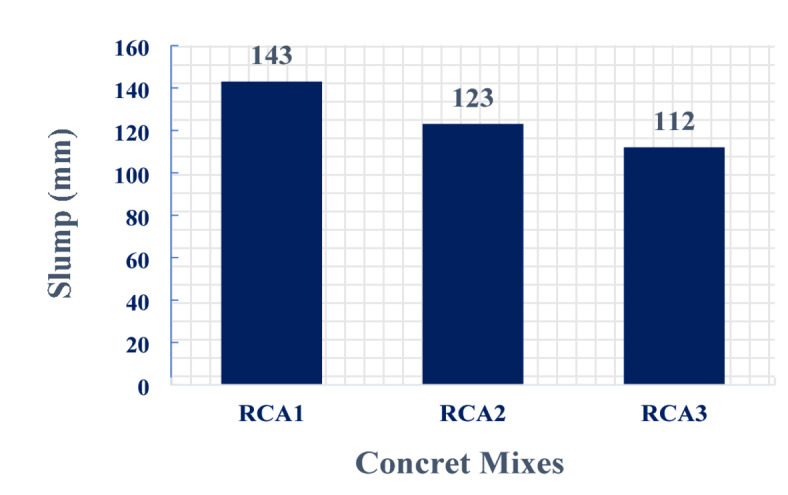
Slump for concrete mixes.

The incorporation of RCA in concrete mixtures typically results in a decrease in slump compared to concrete made with natural aggregates, even when using the same water-cement ratio^32^. This reduction in workability is attributed to the higher water absorption capacity, rougher surface texture, and irregular shapes of RCA particles^33^. Figure 7 illustrates the slump cone test results for three distinct concrete mixtures: the control mix (RCA1) and two modified aggregate gradation mixes (RCA2 and RCA3). The control mix (RCA1) exhibited a slump value of 143 mm, indicative of a relatively fluid consistency. In contrast, the modified mix RCA2, incorporating a 10% #1″ aggregate (S1), displayed a reduced slump of 123 mm. This decrease in slump suggests a stiffer mix, potentially due to the increased proportion of larger aggregate particles, which can hinder the flow of the concrete. Furthermore, the modified mix RCA3, characterized by a with 10% #1″, 10% #4″, and a 10% reduction in #3/8″ aggregate (S2), exhibited a significant reduction in slump, measuring 112 mm. This substantial decrease in slump value is likely attributed to the combined effect of the increased proportion of larger aggregate particles and the reduced proportion of finer aggregate, resulting in a significantly stiffer mix. These findings highlight the significant impact that modifications to the aggregate gradation can have on the workability of concrete. By adjusting the particle size distribution, it is possible to tailor the slump of the concrete mix to specific project requirements. However, it is important to note that while a reduced slump can improve the strength and durability of concrete, it can also make the mix more difficult to place and finish.

### Mechanical properties

**Fig. 8:**
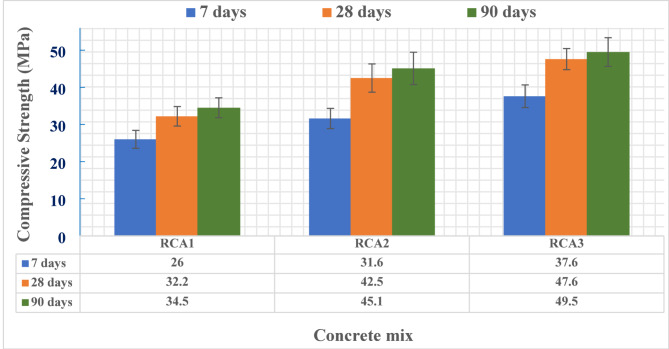
Compressive strength of all designed mixes.

**Fig. 9:**
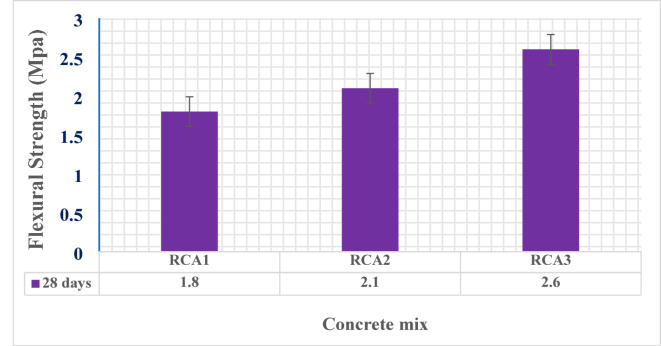
Flexural strength of all designed mixes.

Figures 8 and 9 present a comparative analysis of the mechanical properties of the concrete samples. Specifically, Fig. 8 shows the compressive strength development at 7, 28, and 90 days of curing, while Fig. 9 displays the flexural strength at 28 days. A clear trend emerges: concrete samples incorporating modified aggregate gradations (RCA2 and RCA3) consistently outperformed the control sample RCA1 with the original gradation. Specifically, RCA2 achieved significant improvements in compressive strength, with increases of 21.5%, 32%, and 30.7% at 7, 28, and 90 days, respectively, compared to RCA1. This translates to compressive strengths of 31.6, 42.5, and 45.1 MPa for RCA2, versus 26, 32.2, and 34.5 MPa for RCA1 at the corresponding curing ages. Furthermore, RCA3 demonstrated even more substantial enhancements, reaching compressive strengths of 37.6, 47.6, and 49.5 MPa at 7, 28, and 90 days, respectively. This represents substantial increases of 44.6%, 47.8%, and 43.5% compared to RCA1. These findings underscore the significant influence of modified aggregate gradations on the compressive strength of concrete. The observed increase in compressive strength in RCA2 and RCA3, despite the reduction in slump, can be attributed to the modified aggregate gradation. The specific changes to the gradation likely optimized the particle size distribution, creating a denser and more cohesive mix. This denser packing improves strength but also, counterintuitively, can improve flowability by reducing the surface area requiring lubrication by the cement paste. This allowed to achieve higher strength even with a slightly lower water content, while the optimized packing also contributed to a good workability.

Similarly, the flexural strength at 28 days was consistent with the compressive strength results. Sample RCA2 demonstrated a notable improvement, achieving a flexural strength of 2.1 MPa, a 16.7% increase over the control sample RCA1, which exhibited a flexural strength of 1.8 MPa. Furthermore, sample RCA3 showcased even more significant enhancement, reaching a flexural strength of 2.6 MPa, representing a substantial 44.4% increase compared to the control RCA1 at 28 days of curing. These results underscore the positive impact of modified aggregate gradations on the flexural strength of concrete. The optimized particle size distribution in RCA2 and RCA3 likely contributes to a denser and more interconnected microstructure, resulting in improved resistance to bending stresses. The enhanced performance of RCA2 and RCA3 can be attributed to several factors, including improved particle packing, an enhanced interfacial transition zone (ITZ), and accelerated hydration. The optimized particle size distribution in these modified mixes leads to a more efficient packing of aggregates, reducing voids and increasing the overall density of the concrete. Additionally, the modified gradation may strengthen the ITZ, the critical region between the aggregate and cement paste, leading to improved bond strength and load transfer. The altered particle size distribution could also influence the hydration process, potentially accelerating the formation of C-S-H gel, the primary binding phase in concrete.

Statistical analysis of the obtained mechanical strength data is presented in Table 9. Results show convergence with relatively low values of standard deviation, where the mean value of each three samples with the same mix design and curing age conditions was considered as the final mechanical strength value as presented in Figs. 8 and 9.

**Table 9:** Statistical analysis of strength data.

Sample		Compressive strength value (MPa)			Mean (MPa)	Standard deviation	Coefficient of variation (%)
RCA1	7 days	23.5	28.3	26.2	26.0	2.41	9.25
	28 days	31	30.4	35.2	32.2	2.62	8.12
	90 days	31.4	36	36.1	34.5	2.69	7.78
RCA2	7 days	34.5	31.2	29.1	31.6	2.72	8.61
	28 days	46.2	38.6	42.7	42.5	3.80	8.95
	90 days	49.5	40.8	45	45.1	4.35	9.65
RCA2	7 days	35.5	41.1	36.2	37.6	3.05	8.11
	28 days	50.1	44.5	48.2	47.6	2.85	5.98
	90 days	53.5	49.2	45.8	49.5	3.86	7.80

Sample		Flexural strength value (MPa)			Mean (MPa)	Standard deviation	Coefficient of variation (%)
RCA1	28 days	1.9	1.7	1.8	1.8	0.10	2.00
RCA2		2.1	2.3	1.9	2.1	0.20	9.52
RCA3		2.5	2.8	2.5	2.6	0.17	6.66

### Microstructural properties

#### XRD analysis

**Fig. 10:**
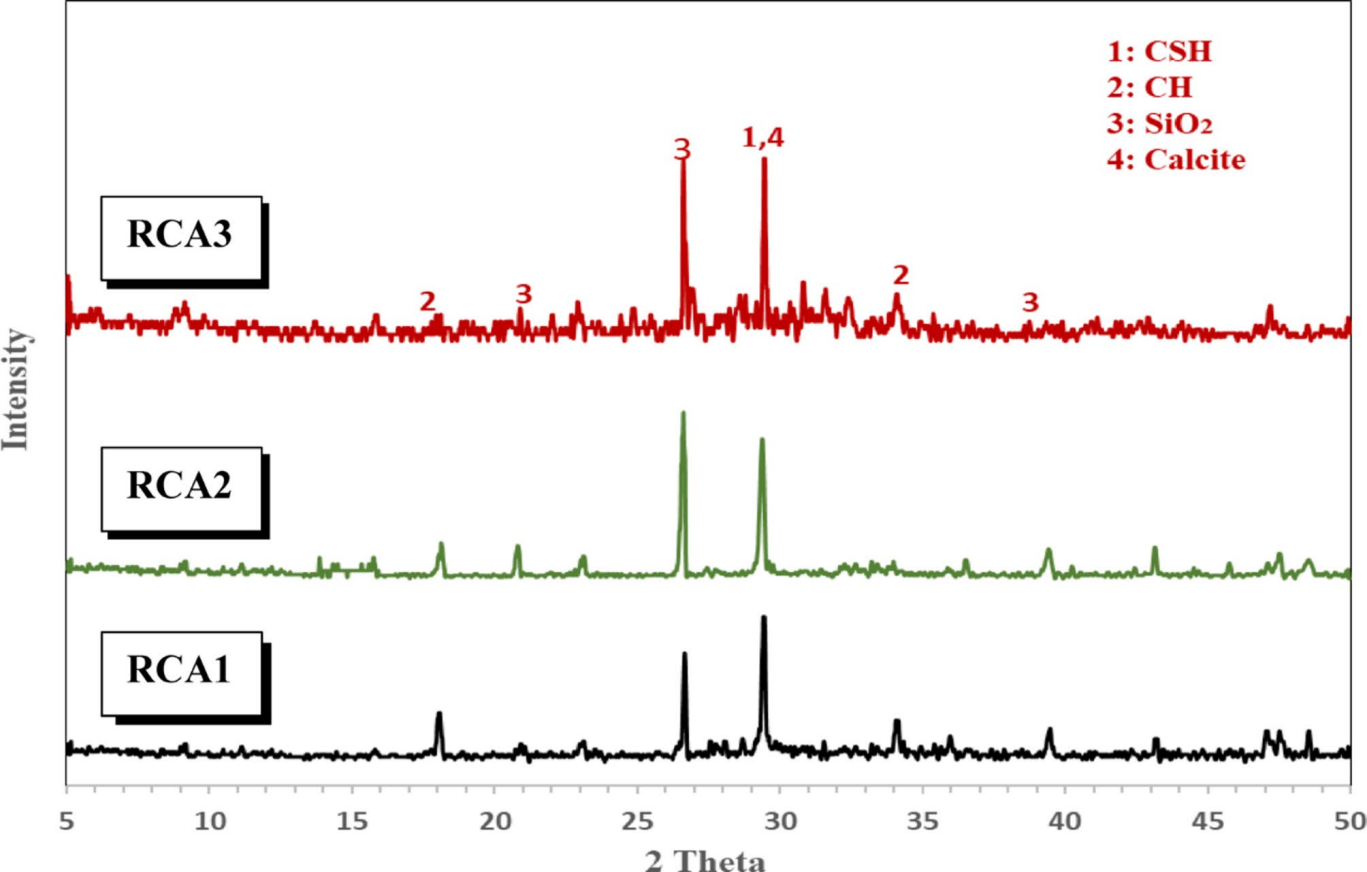
XRD patterns of concrete samples.

XRD analysis is a powerful analytical technique that offers invaluable insights into the intricate microstructure of concrete^34^. XRD analysis is a technique used to study the microstructure, composition, and physical properties of materials. It entails shining X-rays onto a material and studying the diffraction pattern that forms as a result of electrons in the material scattering the X-rays^35^. It is widely employed to investigate the properties and microstructural phases of various substances, including cement, crushed concrete powder, cement paste samples, and additives like nanomaterials and pozzolanic materials^36^. To conduct a comprehensive XRD analysis on concrete samples, rigorous sample preparation is essential. The samples are typically crushed into a fine powder to ensure uniform particle size and optimal X-ray penetration, as mentioned before. Three distinct concrete samples, a control sample (RCA1) and two modified aggregate gradation samples (RCA2 and RCA3), were subjected to XRD analysis at the age of 28 days. The resulting diffraction patterns are presented in Fig. 10 XRD analysis unveiled the presence of several key mineral phases within the concrete samples. The identification of crystalline phases was accomplished using the ICDD PDF database^37^, a comprehensive collection of crystallographic and diffraction data designed for material identification. By analyzing XRD peak positions, which correspond to crystal plane spacing, and applying Bragg’s Law, specific crystal planes were identified. This involved comparing experimental diffraction angles with known values from databases and reference materials^38^. The critical phases include calcium silicate hydrate (CSH, PDF#00-033-0306), a hydration product renowned for its pivotal role in enhancing the strength and durability of concrete, portlandite (CH, PDF#00-004-0733) a byproduct of cement hydration, and calcite (CaCo_3_, PDF#00–005-0586) and quartz (SiO_2_, PDF#01-083-0539), often derived from the aggregate components. It is important to note that calcium silicate hydrate (CSH), the primary binding phase responsible for concrete’s strength and durability, is not a perfectly crystalline compound. Instead, CSH forms a semi-crystalline gel, which does not produce sharp, distinct peaks in XRD patterns. Therefore, rather than referring to “CSH peaks,” we have focused on analyzing the intensity of the broad, diffuse hump in the XRD patterns that is characteristic of CSH gel^39^. Changes in the intensity of this CSH gel hump can be indicative of changes in the amount or degree of CSH formation. Portlandite, a less desirable byproduct of cement hydration, can be converted into more stable phases. Calcite and quartz, derived from aggregates, contribute to the overall composition and physical properties of concrete. Understanding the roles of these phases is crucial for optimizing concrete performance. A comparative analysis of the XRD patterns revealed significant disparities in the crystalline phases between the modified aggregate gradation samples (RCA2 and RCA3) and the control sample RCA1. The XRD analysis suggests that the concrete samples with the modified aggregate gradation sample RCA3 exhibited a more significant impact of the concrete compared to both the control sample with the original gradation RCA1 and the sample with a single modification RCA2. This finding indicates that the combined modifications in RCA3, involving the addition of 10% #1” and 10% #4” aggregates along with a 10% reduction in #3/8” aggregate, led to more pronounced changes in the microstructure and, consequently, the properties of the concrete. The modified aggregate gradation samples (RCA2 and RCA3) exhibited notably higher intensities of the CSH gel hump, indicative of a more advanced stage of hydration. This accelerated hydration process is likely to confer several benefits, including improved mechanical properties, enhanced durability, and a denser microstructure. Conversely, the modified aggregate gradation samples displayed lower peak intensities for CH. This reduction in CH content can be attributed to several factors. Firstly, CH may be consumed in the formation of additional CSH, as evidenced by the increased intensity of the CSH gel hump. Secondly, a decrease in the overall pore volume within the concrete matrix could also lead to a reduced CH content. Both of these factors can positively impact the long-term performance and durability of the concrete by minimizing the risk of deleterious expansion and reducing the susceptibility to various forms of degradation. The modified aggregate gradation samples (RCA2 and RCA3) exhibited enhanced peak intensities for quartz and calcite compared to the control sample (RCA1). This suggests that the altered aggregate gradations influenced the mineral composition of the hardened concrete. Potential factors contributing to this observation include increased quartz content, enhanced carbonation, and mineral dissolution during hydration. While RCA itself does not directly participate in the hydration reactions, the presence of residual mortar on the RCA surface can provide additional nucleation sites for CSH formation^40^. This can potentially influence the overall hydration process, affecting the intensity of the CSH observed in the XRD patterns. The CSH intensity increased for RCA2 and RCA3 with modified aggregation of RCA, likely due to a combination of factors, including improved particle packing density, which enhances the hydration process, and a more interconnected network of nucleation sites from the residual mortar on the RCA, facilitating increased CSH gel formation. The XRD analysis indicates that the concrete sample with the most significant modifications, RCA3, exhibited the most pronounced changes in its microstructure compared to both the control sample RCA1 and the sample with a single modification RCA2. This suggests that the combined modifications in RCA3, involving the addition of 10% #1″ and 10% #4″ aggregates and a 10% reduction in #3/8″ aggregate, had a more substantial impact on the concrete’s properties. Additionally, the XRD analysis revealed that the RCA2 sample, with a 10% increase in #1″ aggregate, also showed changes compared to the control sample. However, these changes were less pronounced than those observed in RCA3. This implies that while a single modification can influence the concrete’s microstructure, multiple modifications, as implemented in RCA3, can lead to more significant alterations. These findings highlight the importance of aggregate gradation in shaping the microstructure and, ultimately, the properties of concrete. By carefully adjusting the aggregate size distribution, it is possible to optimize the performance of concrete in terms of strength, durability, and other desired characteristics.

#### SEM and EDX analysis

**Fig. 11:**
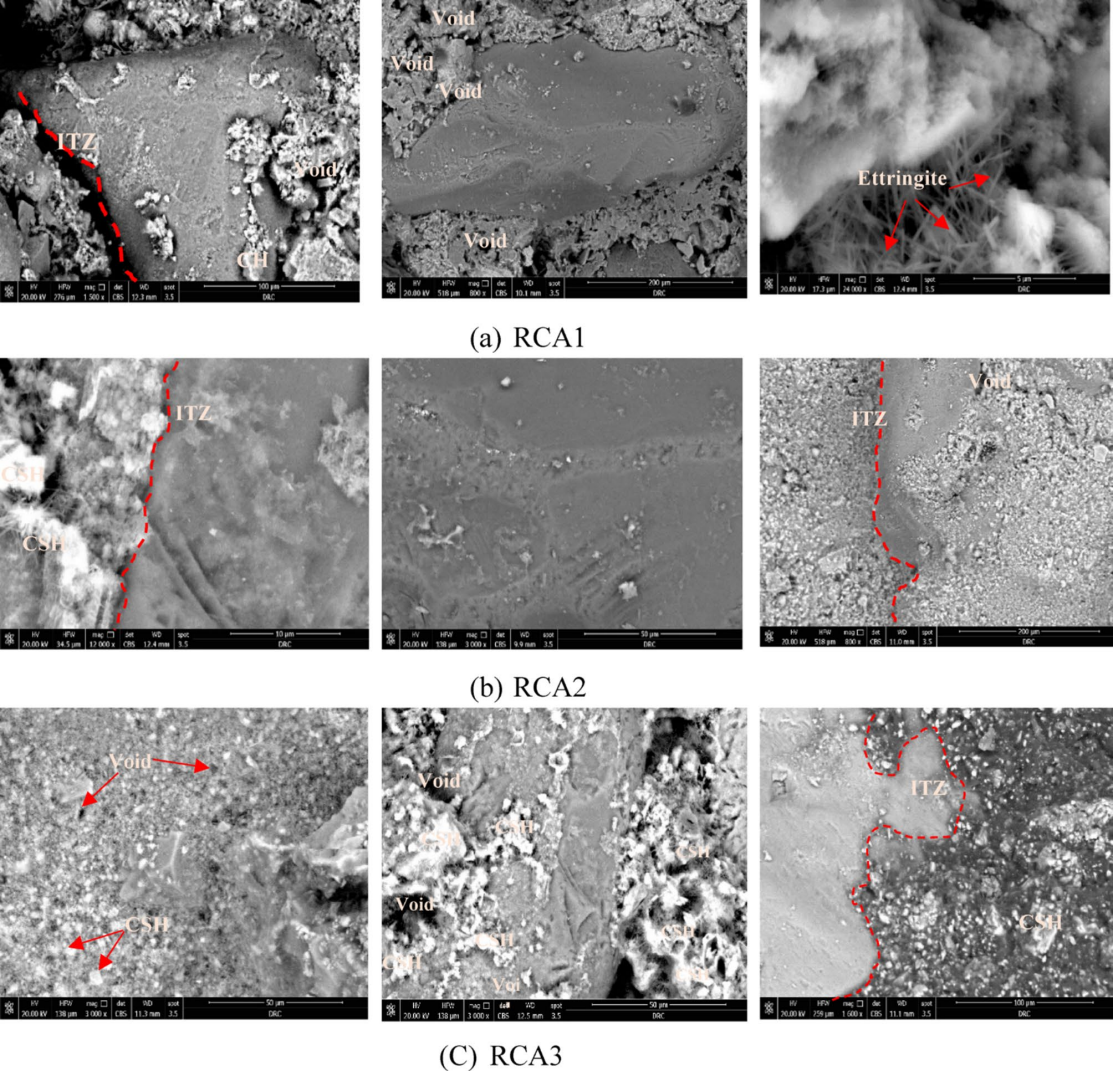
SEM images for RCAX where (**a**) X = 1, (**b**) X = 2, and (**c**) X = 3.

The primary hydration products resulting from sulfate attack on concrete include plate-like portlandite (CH), needle-shaped ettringite, fibrous and gel-like calcium silicate hydrate (C-S-H), and calcium aluminate hydrate (C-A-H)^41^. Subsequent to cement hydration, supplementary phases such as portlandite, calcium silicate hydrates, gypsum, and ettringite develop^42^. Cement particles often fail to blend homogeneously with the other components in fresh concrete, particularly around recycled aggregates. This uneven distribution is exacerbated by the shear forces acting on the cement paste during mixing, which can lead to water separation and the formation of natural aggregates. Consequently, the narrow spaces between recycled aggregates and the cement paste tend to be water-rich and deficient in cement particles compared to the rest of the concrete. As displayed in Fig. 11 SEM analysis revealed distinct microstructural characteristics among the three concrete samples: the control mix (RCA1) and the modified aggregate gradation mixes (RCA2 and RCA3). The control sample exhibited a less homogeneous and less dense cement paste with noticeable voids and microcracks with plenty of long needles ettringite and weak ITZ, indicative of incomplete hydration and a weaker matrix. This suggests potential vulnerabilities to external factors such as moisture penetration and chemical attack. In contrast, both RCA2 and RCA3 displayed denser and more interconnected cement paste matrices with plenty of CSH. The ITZ between the aggregate and cement paste were notably refined in these modified aggregate gradation samples. This refined ITZ suggests enhanced bonding, which can contribute to improved mechanical properties such as compressive strength and tensile strength. Additionally, it can enhance durability by mitigating the risk of interfacial debonding, a common cause of structural failure. The modified aggregate gradation may have created a more favorable environment for the hydration of the cement paste in the vicinity of the aggregate. This could have led to an accelerated formation of CSH, resulting in the observed increase in CSH concentration within the ITZ. The modified RCA gradation may also have influenced the diffusion of ions involved in the hydration process, further facilitating the formation of CSH. In general, RCA gradation indirectly impacts CSH formation. A well-graded RCA can optimize particle packing, influencing water retention, ion transport, and the ITZ, thereby affecting CSH development.

The inherent materials present in RCA, primarily the residual mortar adhered to the aggregate surface, influence both hydration and diffusion processes within the concrete matrix. While this residual mortar does not directly participate in the primary hydration reactions, it can significantly affect the process in several ways. First, the residual mortar provides additional nucleation sites for CSH gel formation, potentially accelerating early hydration and affecting the intensity of the CSH gel hump observed in XRD. Second, this pre-existing, partially hydrated material can alter the local water-to-cement ratio at the RCA-cement paste interface, impacting the kinetics of hydration reactions in that region. Third, the inherent porosity of the residual mortar, coupled with microcracks often present in the RCA itself, can affect water absorption and transport within the concrete matrix. This, in turn, influences both the overall degree of hydration and the diffusion of ions and other chemical species crucial for long-term durability. In general, RCA gradation indirectly impacts CSH formation. A well-graded RCA can optimize particle packing, influencing water retention, ion transport, and the ITZ, thereby affecting CSH development.

**Fig. 12:**
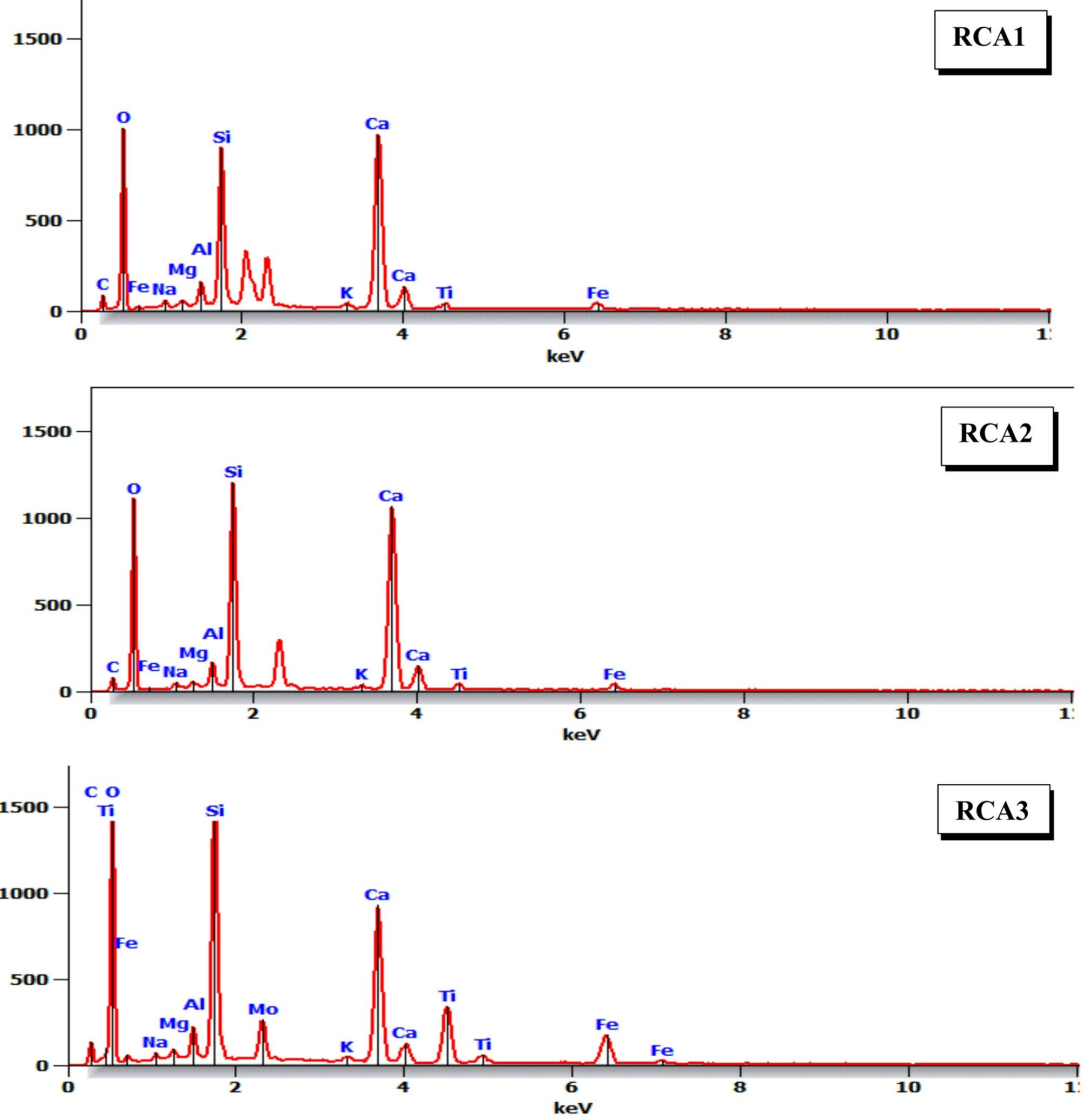
EDX patterns for concrete samples.

To gain a deeper understanding of the elemental composition of the concrete samples, EDX analysis was conducted^43^. This technique focused on oxygen, calcium, and silicon, as these elements are fundamental constituents of hydration products, primarily C-S-H gels. By analyzing the elemental spectra, researchers can assess the relative abundance of these elements and draw inferences about the degree of hydration, the formation of specific hydration products, and the overall quality of the concrete. EDX analysis revealed that the presence of elevated calcium and silica concentrations within the concrete samples is indicative of the formation of C-S-H gel, a crucial hydration product responsible for the development of concrete strength and durability. By examining the elemental composition, particularly the calcium-to-silicon (Ca/Si) ratio, valuable insights into the microstructure and potential performance of the concrete can be gained. A lower Ca/Si ratio is often associated with a higher degree of C-S-H polymerization^44^, leading to the formation of denser and more robust C-S-H structures. This enhanced C-S-H network contributes to improved mechanical properties, including compressive strength and tensile strength^45,46^. However, it is important to note that the Ca/Si ratio is just one factor influencing concrete performance, and a comprehensive analysis of microstructural and mechanical properties is necessary for a complete understanding. EDX analysis of the three concrete samples (RCA1, RCA2, and RCA3) revealed variations in their elemental composition, as shown in Fig. 12. The calculated Ca/Si ratios for these samples, detailed in Table 10, were 2.08, 1.93, and 1.11, respectively. Previous studies have shown that a lower Ca/Si ratio improves the polymerization of C-S-H and, consequently, the performance of concrete^47^. The lower Ca/Si ratios observed in RCA2 and RCA3 are consistent with these findings. This enhanced polymerization is likely due to sufficient dissolution of the cementitious material within the concrete matrix^48^. This finding is significant as it indicates that the modified aggregate gradation in RCA2 and RCA3 may have influenced the hydration process, leading to the formation of a more favorable C-S-H microstructure. While the Ca/Si ratio provides valuable information about the degree of C-S-H polymerization, it is essential to consider other factors that may influence concrete performance. These factors include the porosity of the concrete, the distribution of hydration products, and the interfacial transition zone between the aggregate and the cement paste.

**Table 10:** EDX results for different RCAX concrete mixes.

Mix	Density ρ, (g/cm^3^)	O K	Ca K	Si K	Al K	Fe K	K K	Na K	C K	Ti K	Ca/Si Ratio
RCA1	2.31	46	29.5	14.2	1.8	2.7	0.6	0.7	2.5	1.3	2.08
RCA2	2.39	45.8	29.4	15.2	1.6	2.9	0.4	0.6	2.3	1.5	1.93
RCA3	2.42	45.8	16.7	15	1.4	10	0.4	0.6	2.7	7.4	1.11

### Radiation shielding results

**Fig. 13:**
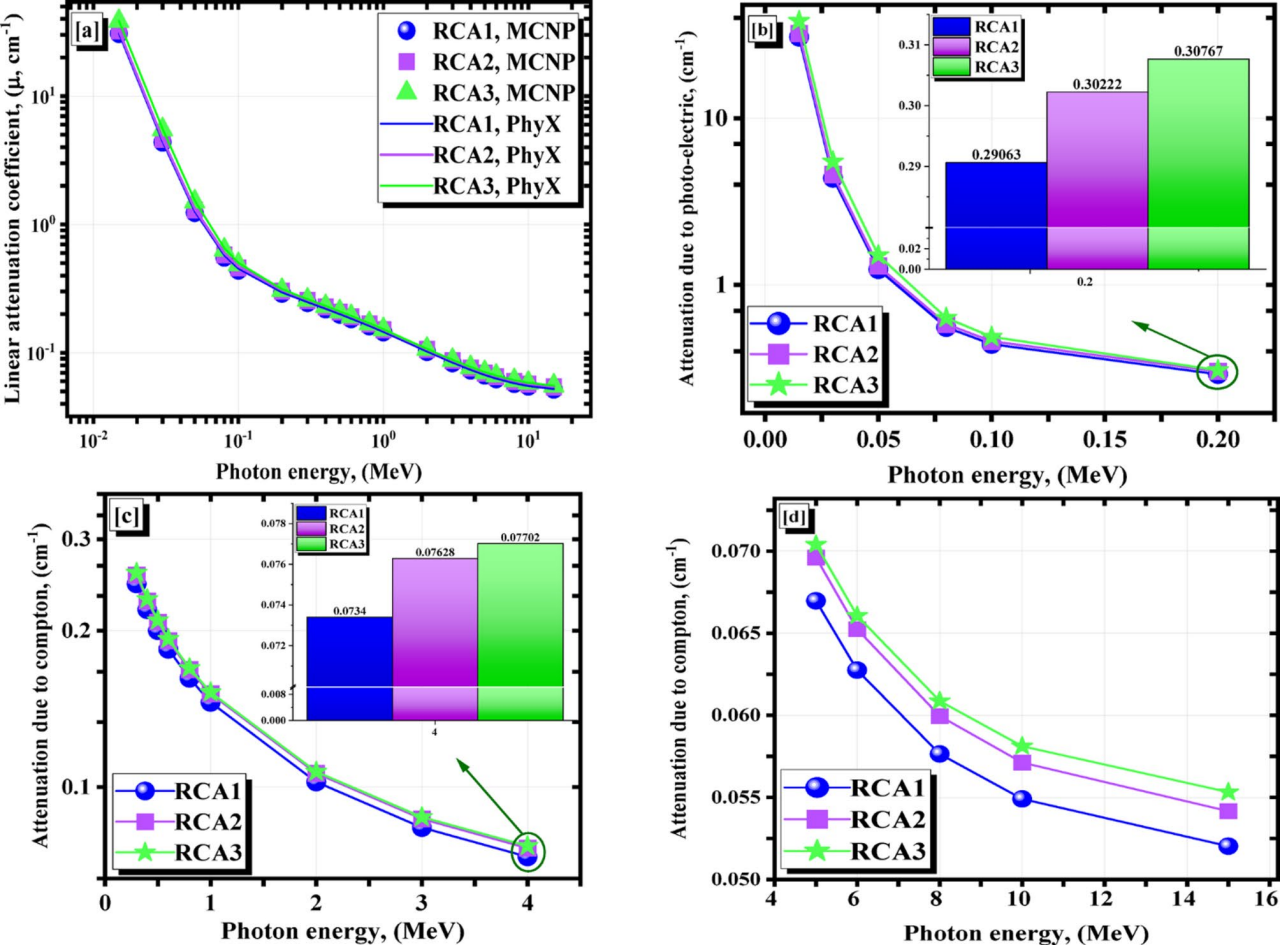
Impact of γ-ray on µ of (**a**) obtained from MCNP and PhyX, (**b**) photo-electric, (**c**) and (**d**) compton scattering for the investigated RCAX concrete samples.

Before investigating the computed values of the various γ-rays’ shielding parameters, a comparison between the calculated (µ) values within a wide investigated energy range using both PhyX software and the model created by MCNP^49^ has been performed using EDX analysis results. The simulated µ values at all studied γ-rays’ energies are in excellent agreement with those calculated by the PhyX as seen in Fig. 13a. Figure 13b–d represent the µ’s behavior against the γ-energies. There were two trends of the µ’s decreasing as follows:

(A) the trend due to the photo-electric effect, which the cross-section (q) was proportional to the γe^− 4^. The increase of the applied γe values between γe ≤ 0.200 MeV causes a tough exponential decreasing tendency. The µ values were from 30.788 to 0.291 cm^− 1^ for RCA1, from 32.299 to 0.302 cm^− 1^ for RCA2, and from 38.353 to 0.308 cm^− 1^ for RCA3 sample at γe ≤ 0.200.
(B) the trend due to the Compton effect, in which the q α γe^− 1^. The improvement in γe values was associated with a gradual reduction in the q as the quantity of γ-electron interactions decreased, and then with a gradual decrease in the µ values. At 0.0300 ≤ γe ≤ 15, the µ values for RCA1 ranged from 0.246 to 0.052 cm^− 1^, for RCA2 were 0.256 to 0.054 cm^− 1^, and for the RCA3 sample, were 0.259 to 0.055 cm^− 1^.

**Fig. 14:**
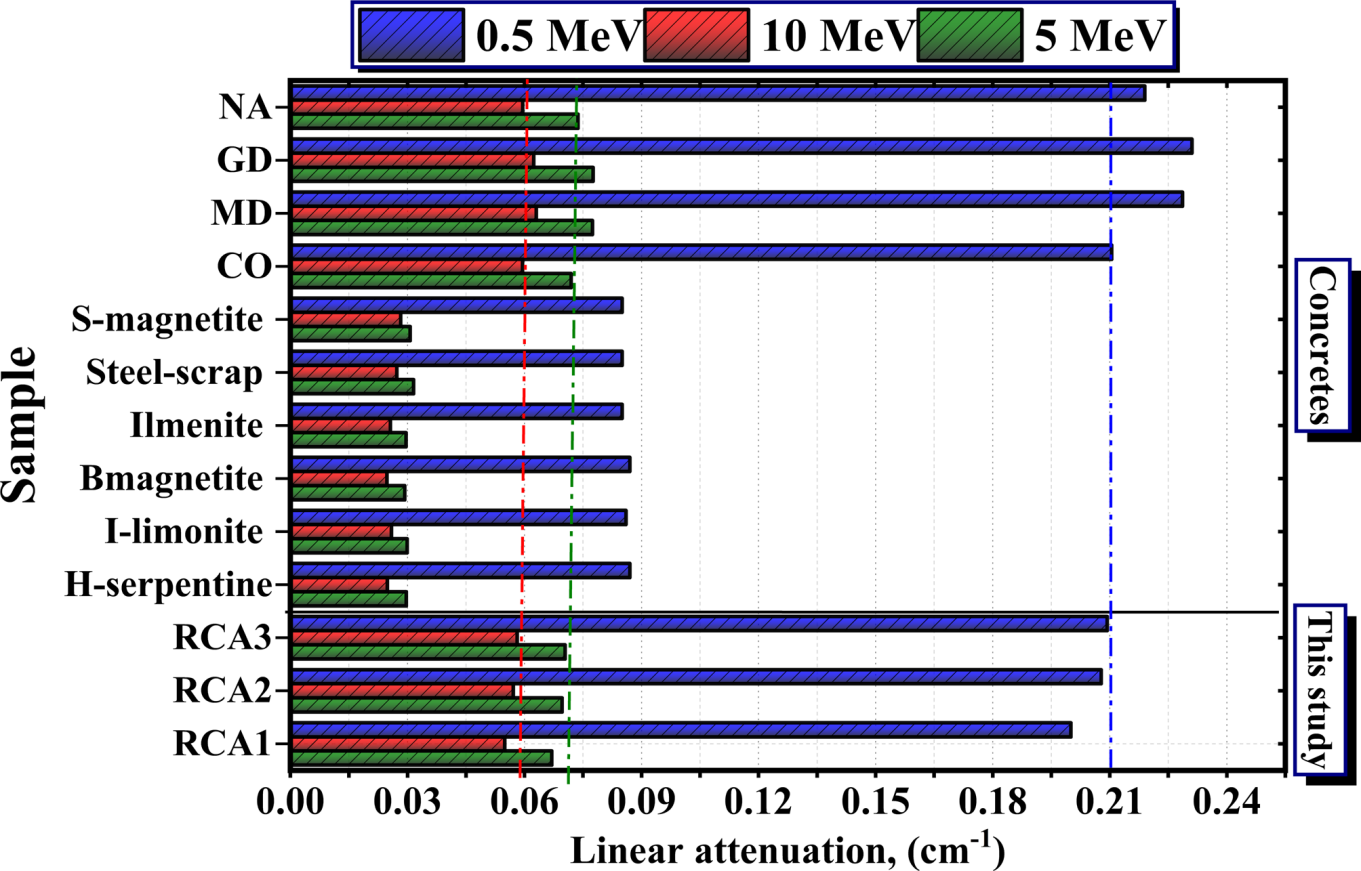
Comparison between the *µ* vs. the γ-energy for the investigated RCAX concrete samples with other published work.

Figure 14 lists the linear attenuation values for the RCAX concrete samples and some other concretes (H-serpentine, I-limonite, B-magnetite, Ilmenite, Steel-scrap, and S-magnetite) and set concrete samples of waste concrete; Marble (MD), granite (GD), and nano alumina (NA) at selected energies 0.5, 5, 10 MeV^43^. At 0.5 and 10 MeV, the RCA3 sample was found higher than those compared except MD, GD, and NA. At 5, the RCA3 sample was found higher than those compared except MD, and GD.

From the results, the RCA3 sample possesses the highest values, of the linear attenuation, due to the high-Z alloying elements such as Fe and Ti (10%, and 7.4%) as well as the high density (2.420 g/cm^3^).

**Fig. 15:**
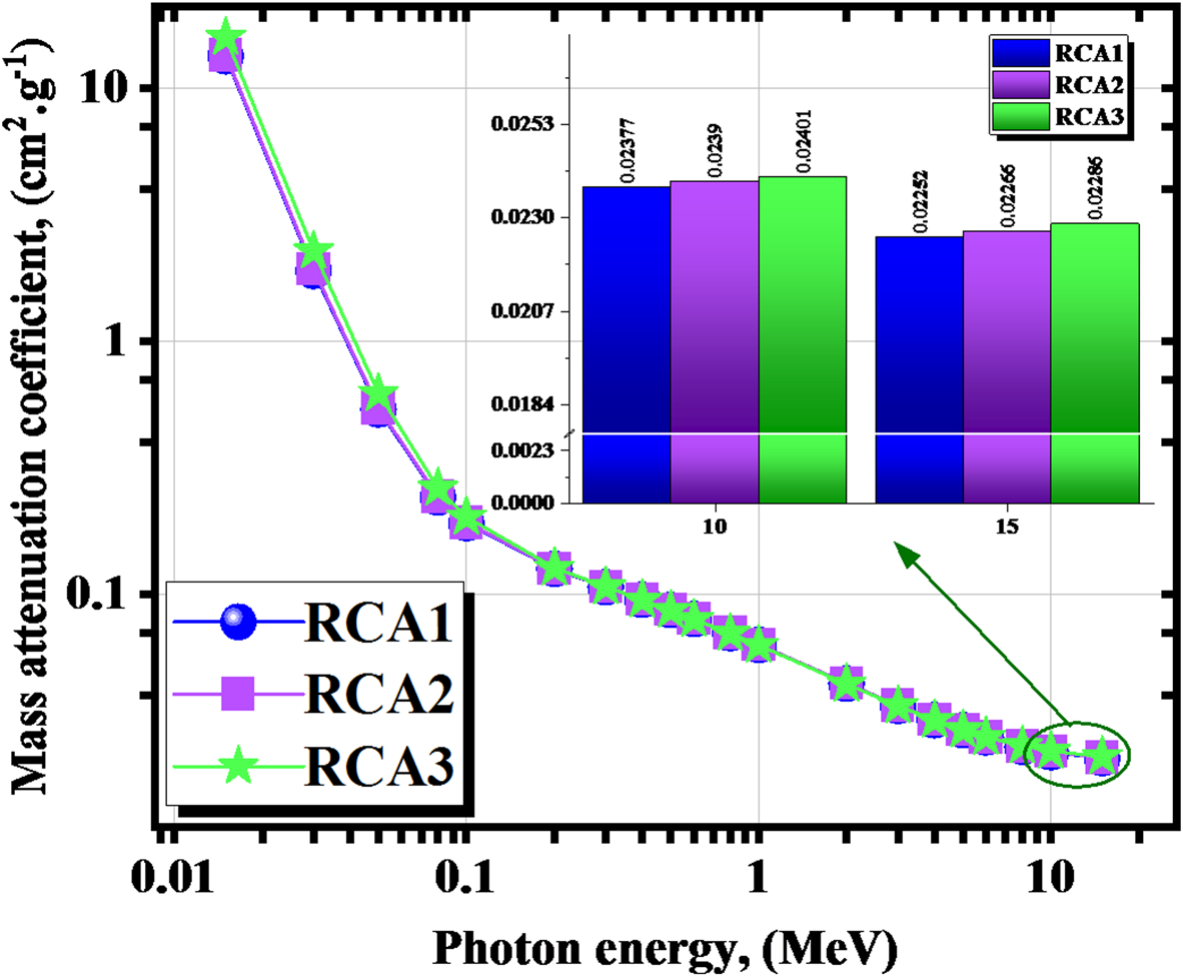
The *µ*_*c*_ vs. the γ energy for the investigated RCAX concrete samples.

Figure 15 represents the µ_c_’s behavior against the γ-energies. The µ_c_ values were from 13.328 to 0.023 cm^2^.g^− 1^ for RCA1, from 13.514 to 0.023 cm^2^.g^− 1^ for RCA2, from 15.849 to 0.023 cm^2^.g^− 1^ for RCA3 sample at 0.015 ≤ γe ≤ 15.

**Fig. 16:**
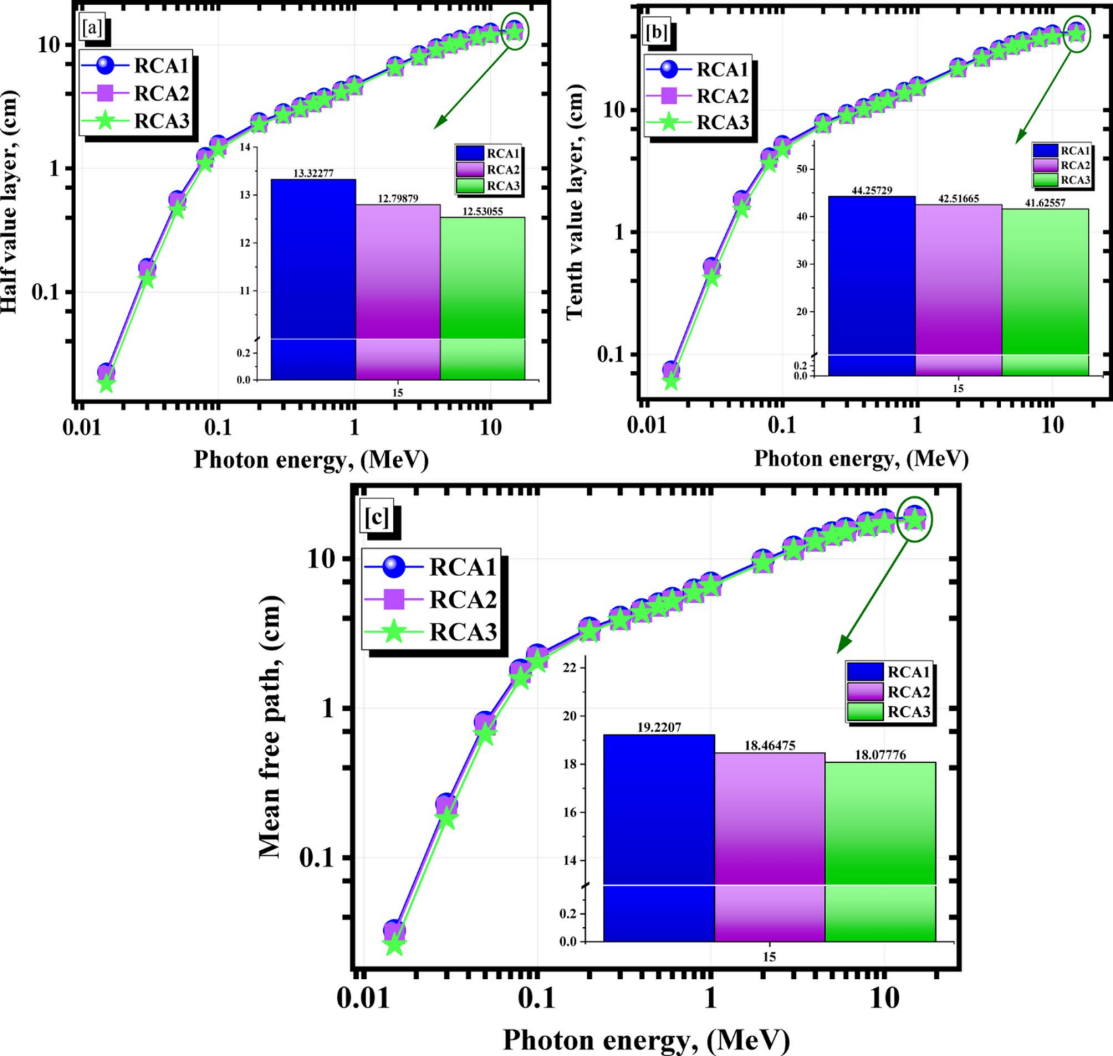
(**a**) HV, (**b**) TV, and (**c**) MF for the investigated RCAX concrete samples vs. γ-energy.

The required shield thicknesses to attenuate are 50%, 90%, and about 67% of the incident photons, which are HV, TV, and MF, respectively, are presented in Fig. 16a–c for the studied natural samples. The HV values were from 0.023 to 13.323 cm for RCA1, from 0.021 to 12.799 cm for RCA2, and from 0.018 to 12.531 cm for RCA3 sample at 0.015 ≤ γe ≤ 15 (Fig. 16a). Also, the TV values were from 0.075 to 44.257 cm for RCA1, from 0.071 to 42.517 cm for RCA2, and from 0.060 to 41.626 cm for RCA3 sample at 0.015 ≤ γe ≤ 15 (Fig. 16b). The MF parameter has the same trend (Fig. 16c).

**Fig. 17:**
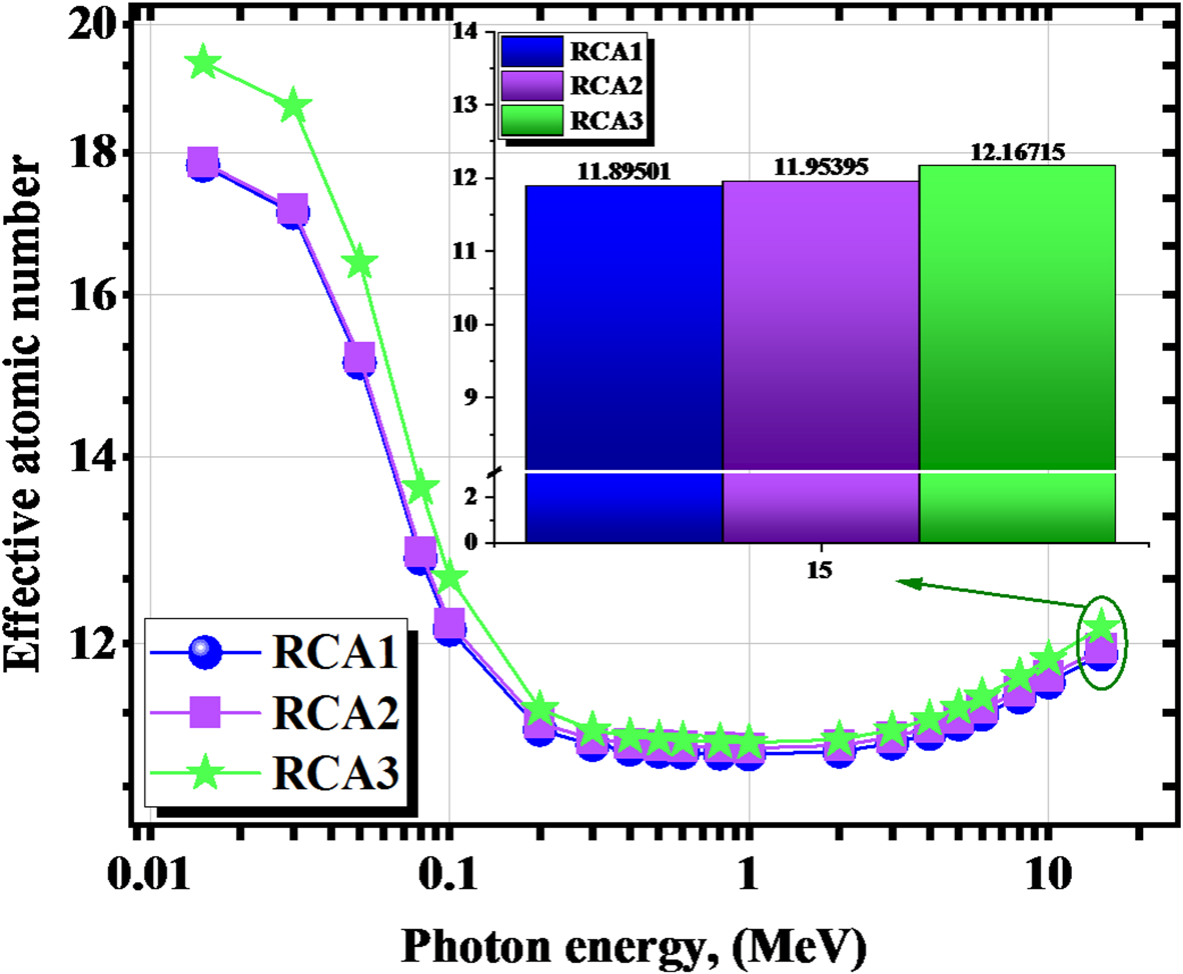
The effective atomic number (Z_eff_) for the investigated RCAX concrete samples versus γ-energy.

Considering the effective atomic numbers (Z_eff_) and µ values computed for the studied RCAX concrete samples within the energy range of interest γe ≤ 15, Fig. 17 depict the interrelation between the Z_eff_ shielding parameter and γ-energy. Based on the results shown in the previous figures, Z_eff_ is known to be attributed to the γ-rays interaction modes with the attenuating medium; its value usually varies with the γ-energy^50^. As a consequence of this, higher values are observed at low energies as a result of the control of the γ-electric mechanism, which is significantly dependent on the Z_eff_ of the shield constituents. On the other hand, for the current energy range that was studied, which ranged from 0.200 ≤ γe ≤ 15 MeV, the lowest values were observed throughout the majority of this range, with the exception of the onset, and they were almost independent of the γ-energy that was incident. That can be attributed to the dominancy of the Compton scattering mechanism at these energies. The Z_eff_ values were from 17.808 to 11.895 for RCA1, from 17.849 to 11.954 for RCA2, from 19.386 to 12.167 for RCA3 sample at γe ≤ 15.0.

**Fig. 18:**
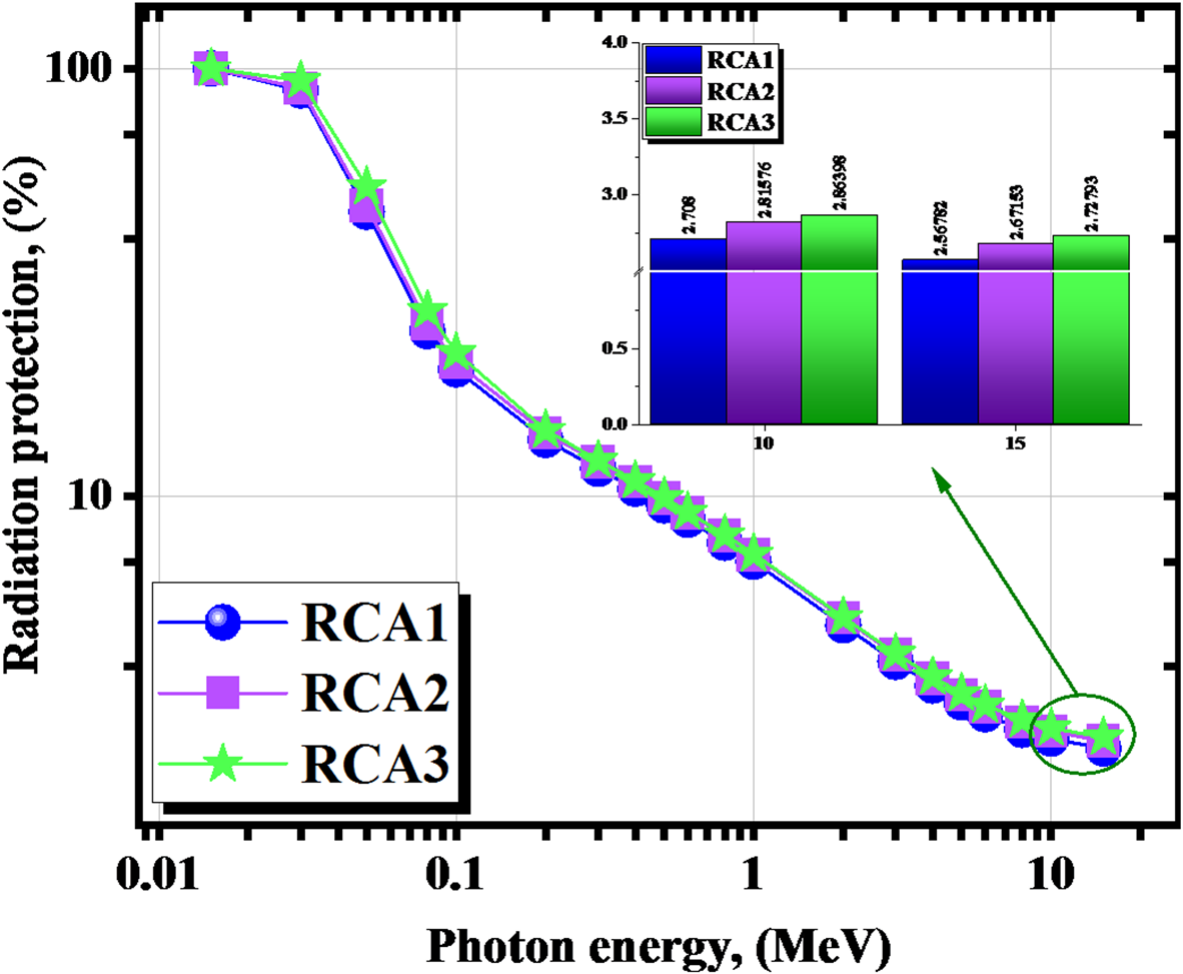
The RP for the investigated RCAX concrete samples versus γ-energy.

As mentioned before, RP is an essential statistical parameter that should be taken into account when determining the level of attenuation that the shield could provide^35^. Figure 18 shows that the RP values are more than 19% at γe ≤ 0.1 MeV. When the incident γ-energy increases, the penetration power of the incident γs also increases, leading to a weighty decrease in the RP (%) levels. Therefore, at the start of the studied γ-energy range, the superiority of the RCA3 sample over the other samples, thus, its γ-rays’ shielding efficiency, can be considered tangible and effective while dealing with low γ-rays’ radiation fields. The RP values dropped from about 19.779, 20.525, 21.664, and 20.985% at 0.100 MeV to only 2.568, 2.672, and 2.728%, respectively, for all RCA1, RCA2, and RCA3 at γe = 15 MeV.

**Fig. 19:**
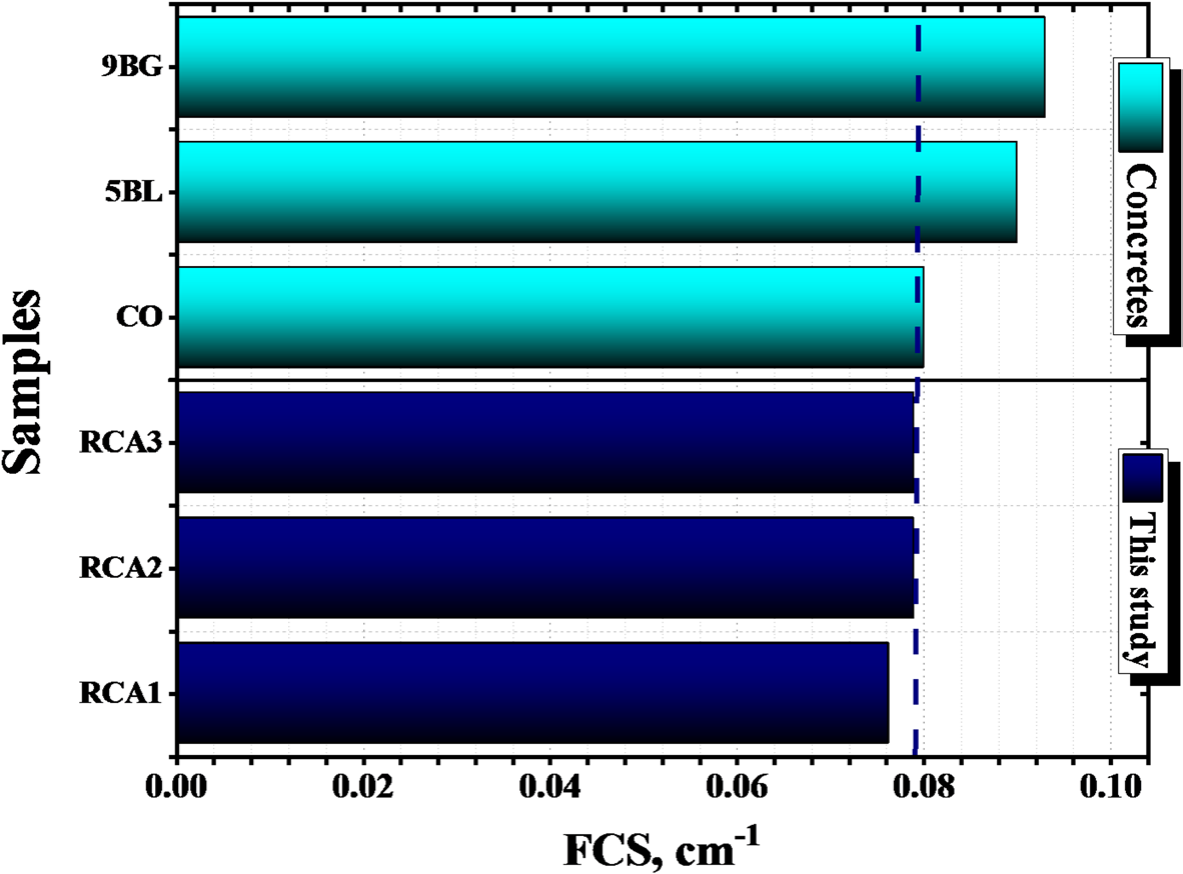
Comparison of The FCS for the investigated RCAX concrete samples and some concrete samples.

**Fig. 20:**
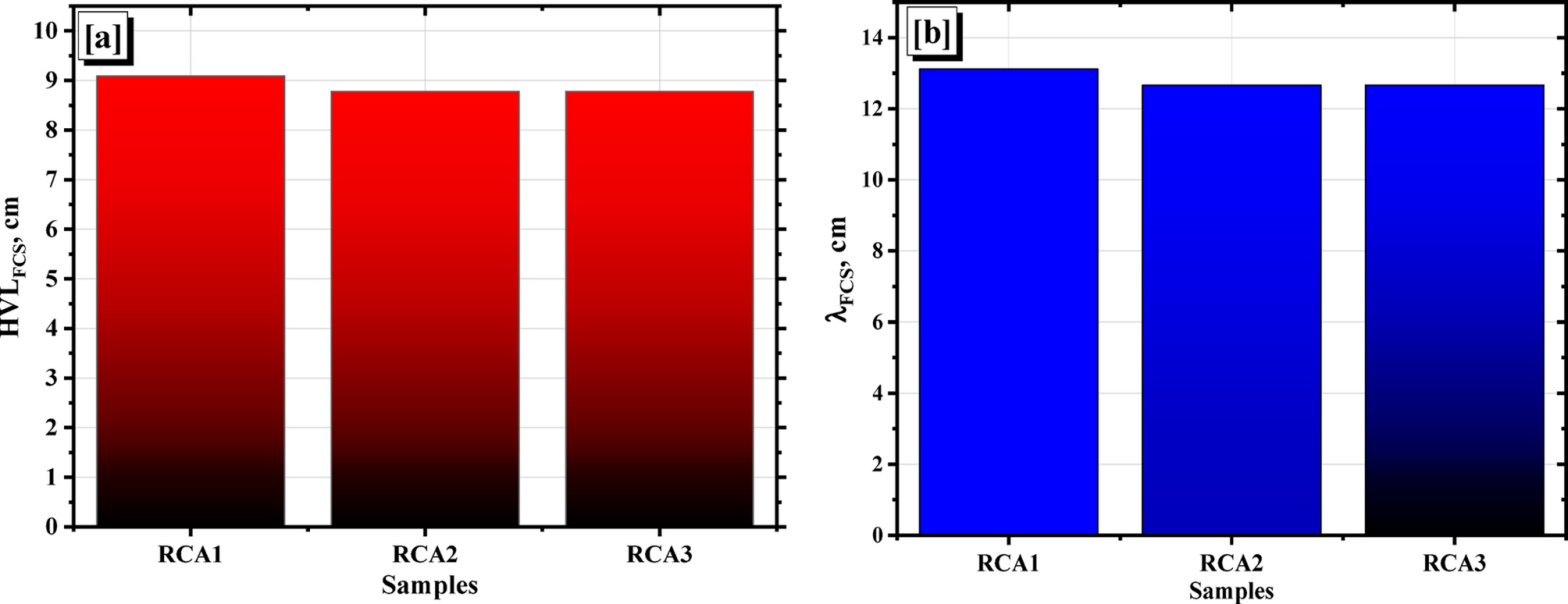
(**a**) The HVL_FCS_, and (**b**) the λ_FCS_ for the investigated RCAX samples.

The neutron shielding of a material is one of the parameters that determines the extent of the material’s ability to block fast neutrons, by calculating the FCS, HVL_FCS_, and λ_FCS_. Figure 19 represents a comparison of the FCS for the investigated samples with some previews published concrete samples^35^. Figure 20 represents the HVL_FCS_, and the λ_FCS_ for the investigated samples. The RCA2, RCA3 samples were in first place with average Σ_R_ equals 0.079 cm^− 1^ and corresponding average HVL_FCS_ and λ_FCS_ equal 8.780 and 12.667 cm, respectively. In contrast, RCA1 has the lower Σ_R_ value (0.076 cm^− 1^) and the most significant thicknesses for both HVL and λ, equal to 9.093 and 13.118 cm, respectively. Possessing the high content of low-Z elements like carbon (2.30, 2.70 wt%) and the high density (2.390, 2.420 g/cm^3^) can be considered the reason for putting RCA2, RCA3 samples, respectively, in the lead regarding attenuating fast neutrons that can be achieved by increasing neutrons and sample interactions depending mainly on the inelastic scattering mechanism.

## Conclusions

Recent research has focused on the potential of recycled concrete aggregate (RCA) as a sustainable construction material. This study assessed the suitability of RCAs derived from North Sinai materials. Three concrete mixes were investigated: a control mix (RCA1) with 100% original gradation of recycled aggregate, and two modified mixes: RCA2 (10% #1″ improved gradation (S1)), and RCA3 (10% # 1″ + 10% #4″ + Reduction10% # 3/8″ (S2)). The research focused on the effects of modified gradation on the fresh properties (workability) and mechanical properties (compressive and flexural strengths), and microstructure (analyzed using XRD, SEM and EDX). Additionally, the study investigated the concrete’s resistance to different types of radiation, including its ability to attenuate γ-rays and neutrons. Key findings from this research are summarized as follows:

1. The slump cone test results clearly demonstrate that modifications to the aggregate gradation significantly impact concrete workability. The control mix (RCA1) exhibited a relatively high slump, indicating a fluid consistency. In contrast, the modified aggregate gradation mixes, RCA2 and RCA3, showed substantial reductions in slump. This decrease is attributed to the modification in the proportion of aggregate particle sizes, which influences the flowability of the concrete mix.
2. RCA2 and RCA3, exhibited significant improvements in both compressive and flexural strength compared to the control mix, RCA1. According to compressive strength, RCA2 demonstrated increase of 21.5%, 32%, and 30.7% at 7, 28, and 90 days, respectively. RCA3 further outperformed, with increases of 44.6%, 47.8%, and 43.5% at the respective curing ages. Similarly, in flexural strength, RCA2 showed a notable 16.7% improvement over the control mix, while RCA3 achieved a remarkable 44.4% increase at 28 days.
3. XRD analysis revealed that the concrete samples with modified aggregate gradations, significantly impacted the concrete’s microstructure. RCA2 and RCA3 exhibited the most pronounced changes, leading to accelerated hydration, reduced portlandite content, and increased C-S-H formation.
4. SEM analysis revealed that the modified aggregate gradation mixes (RCA2 and RCA3) exhibited superior microstructural characteristics compared to the control mix (RCA1). RCA2 and RCA3 showed denser and more interconnected cement paste matrices with abundant C-S-H and refined ITZs. The modified aggregate gradation likely created a more favorable environment for hydration, leading to improved C-S-H formation and stronger bonding between the aggregate and cement paste. These microstructural enhancements contribute to the improved mechanical properties and durability of the modified concrete mixes.
5. EDX analysis revealed variations in the elemental composition of the three concrete samples. The lower Ca/Si ratio in RCA2 and RCA3 was associated with enhanced C-S-H polymerization, leading to improved concrete mechanical properties compared to RCA1.

Also, this work examines the γ/neutron shielding properties of the RCAX concrete samples. Key findings from this research are summarized as follows:

6. The RCAX’s µ order is RCA1 < RCA2 < RCA3.
7. The RCA3 sample have the lowest HV, TV, and MF.
8. The high performance the RCA3 sample against gamma radiation was due to the high content of Fe and Ti element.
9. RCA’s Z_eff_ changes: 17.808 to 11.895 for RCA1, from 17.849 to 11.954 for RCA2, from 19.386 to 12.167 for RCA3 concrete sample.
10. The RCA2, RCA3 samples were found a promising samples for neutron attenuation.
11. The results indicate that the studied RCAX-concrete samples provide the highest degree of protection against γ-rays and fast neutrons. It could be used in the radiation shielding applications (e.g., hospitals and nuclear power plants, etc.).

The findings suggest that the modified RCA concrete, particularly RCA3, holds promise for sustainable radiation shielding applications in facilities such as hospitals and nuclear power plants due to its enhanced mechanical properties and radiation attenuation capabilities. Future research could explore optimizing the gradation further for specific radiation types or incorporating other waste materials to enhance performance and sustainability. Further studies could also investigate the long-term durability and performance of this modified RCA concrete under various environmental conditions, expanding its potential applications in diverse construction projects.

## Electronic supplementary material

Below is the link to the electronic supplementary material.

Supplementary Material 1

## Data Availability

All data generated or analyzed during this study are included in this published article.
