# Supplementary material for: Impact of modified aggregate gradation on the workability, mechanical, microstructural and radiation shielding properties of recycled aggregate concrete
Source: Sci Rep. 2025 May 26;15:18428. doi: 10.1038/s41598-025-02655-y (PMC12106813; doi:10.1038/s41598-025-02655-y)
Supplement: Supplementary file 1 — Supplementary Material 1 [file 41598_2025_2655_MOESM1_ESM.docx]

The following supplementary information provides additional figures that provide further visualization related to the main manuscript, which are not essential for the core understanding of the presented work but are provided here for completeness and further reference.

| 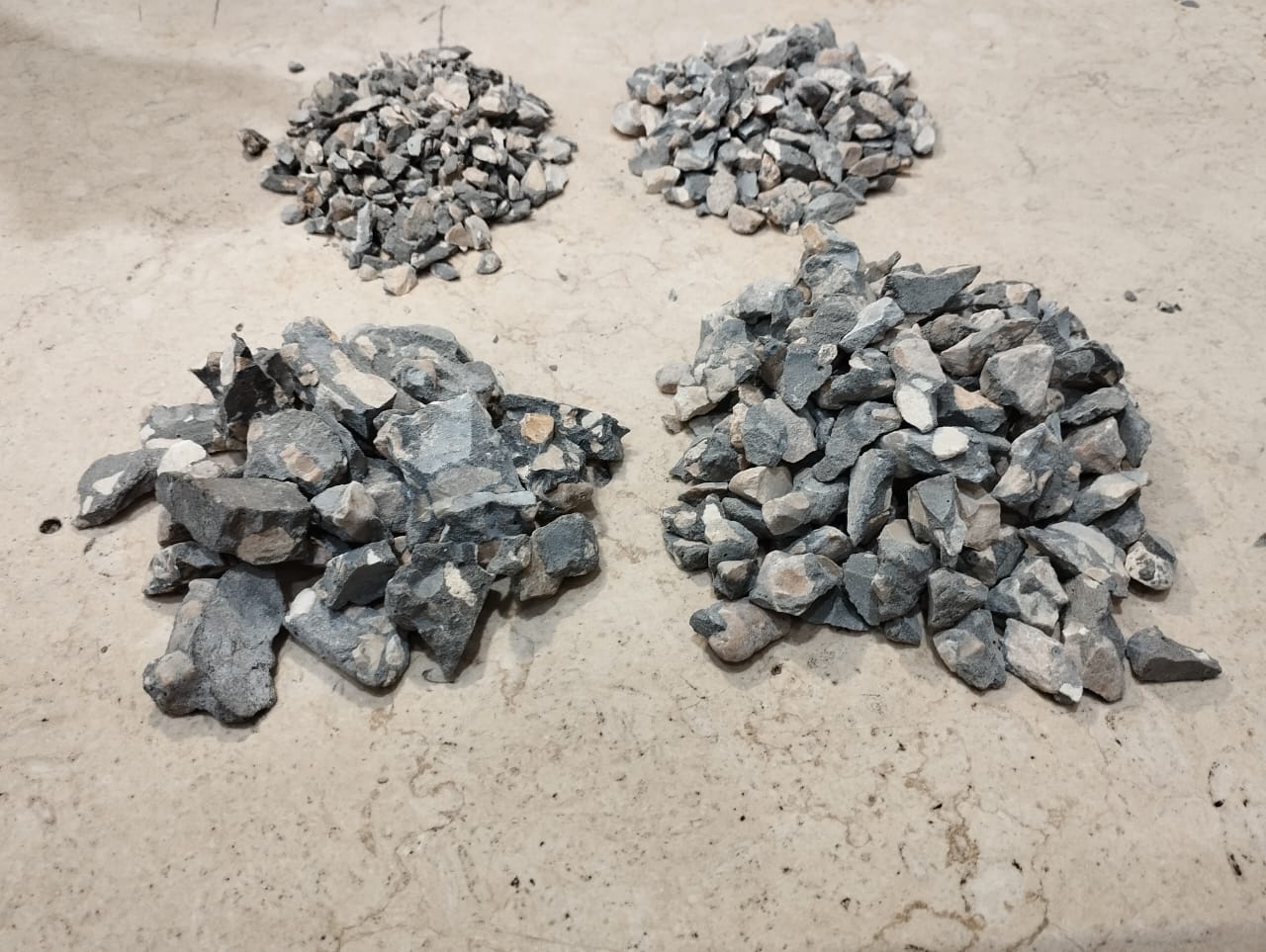 |
| --- |
| **Fig. 1:** Procedure for modifying RCA gradation |
| 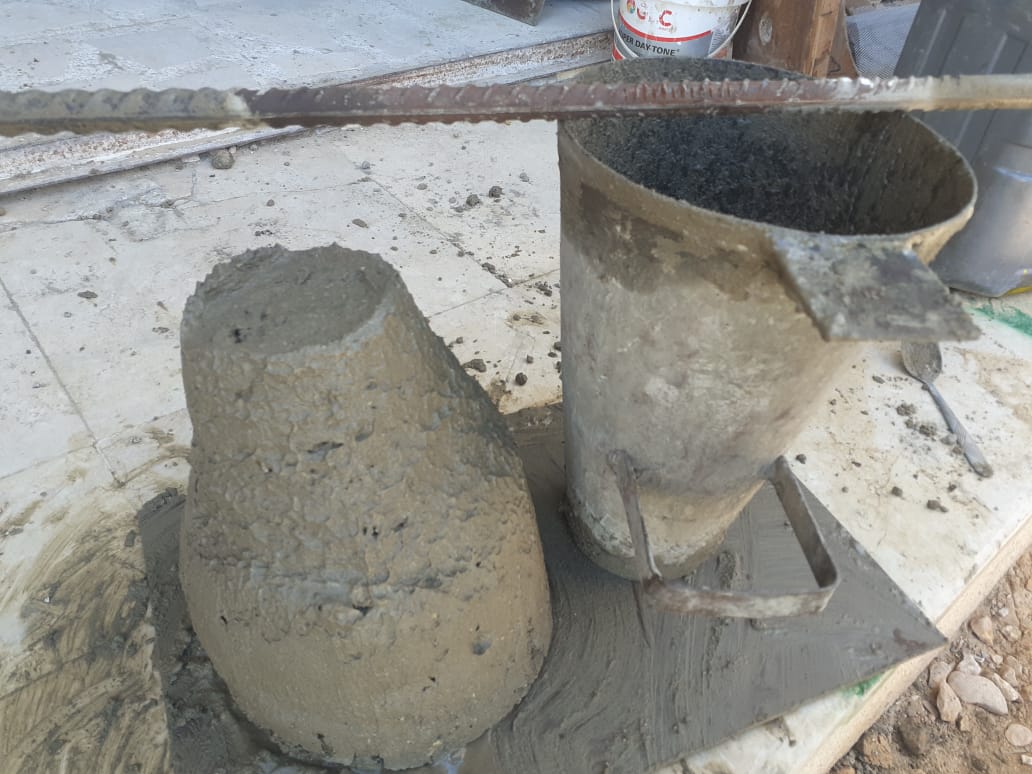 |
| **Fig. 2:** Slump cone test |
| 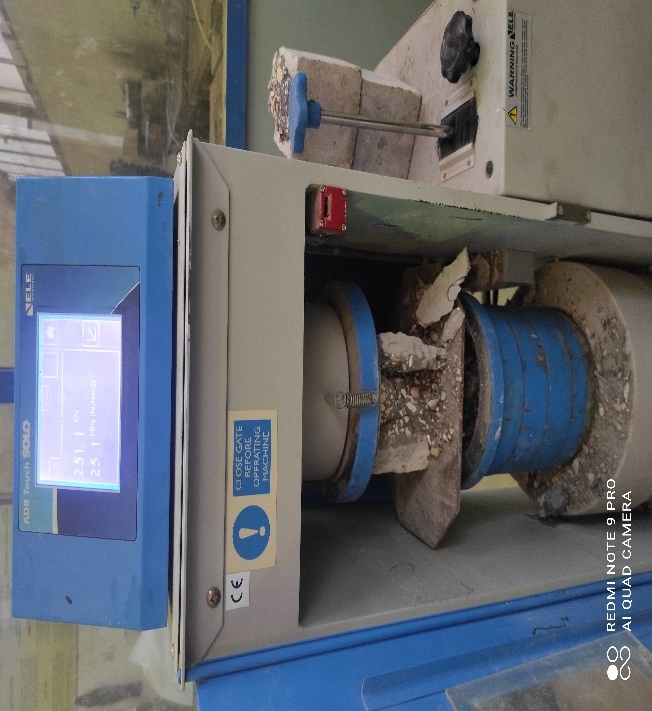 |
| **Fig. 3:** Compressive strength test |

| **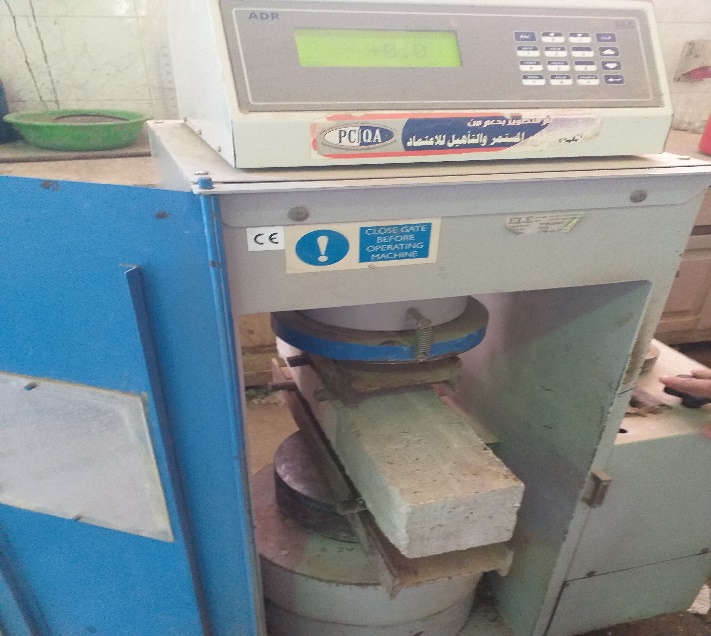** |
| --- |
| **Fig. 4:** Flexural strength test |

| **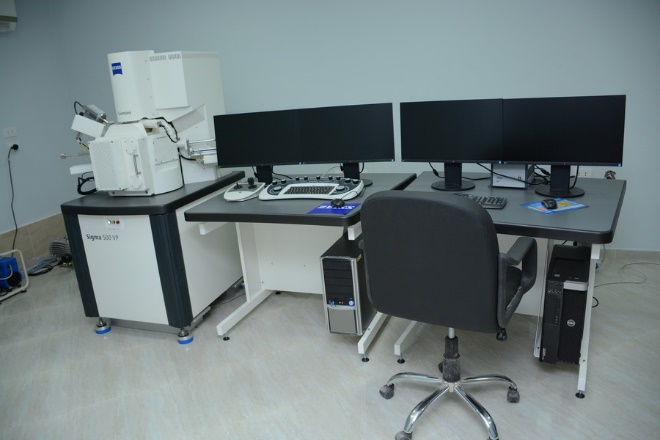** |
| --- |
| **Fig. 5:** SEM device |
